# Mesenchymal Stromal Cells Mediate Clinically Unpromising but Favourable Immune Responses in Kidney Transplant Patients

**DOI:** 10.1155/2022/2154544

**Published:** 2022-02-15

**Authors:** Urvashi Kaundal, Raja Ramachandran, Amit Arora, Deepesh B. Kenwar, Ratti Ram Sharma, Ritambhra Nada, Mukut Minz, Vivekanand Jha, Aruna Rakha

**Affiliations:** ^1^Department of Translational and Regenerative Medicine, Postgraduate Institute of Medical Education and Research, Sector 12, Chandigarh, India; ^2^Department of Zoology, Panjab University, Sector 14, Chandigarh, India; ^3^Department of Nephrology, Postgraduate Institute of Medical Education and Research, Sector 12, Chandigarh, India; ^4^Department of Medical Microbiology, Postgraduate Institute of Medical Education and Research, Sector 12, Chandigarh, India; ^5^Department of Renal Transplant Surgery, Postgraduate Institute of Medical Education and Research, Sector 12, Chandigarh, India; ^6^Department of Transfusion Medicine, Postgraduate Institute of Medical Education and Research, Sector 12, Chandigarh, India; ^7^Department of Histopathology, Postgraduate Institute of Medical Education and Research, Sector 12, Chandigarh, India; ^8^Fortis Hospital, Mohali, India; ^9^The George Institute for Global Health, New Delhi, India

## Abstract

**Background:**

Allograft rejection postkidney transplantation (KTx) is a major clinical challenge despite increased access to a healthcare system and improvement in immunosuppressive (IS) drugs. In recent years, mesenchymal stromal cells (MSCs) have aroused considerable interest in field of transplantation due to their immunomodulatory and regenerative properties. This study was aimed at investigating safety, feasibility, and immunological effects of autologous MSCs (auto-MSCs) and allogeneic MSCs (allo-MSCs) as a complement to IS drug therapy in KTx patients.

**Methods:**

10 patients undergoing KTx with a living-related donor were analysed along with 5 patients in the control group. Patients were given auto-MSCs or allo-MSCs at two time points, i.e., one day before transplant (D-0) and 30 days after transplant (D-30) at the rate of 1.0-1.5 × 10^6^ MSCs per kg body weight in addition to immunosuppressants. Patients were followed up for 2 years, and 29 immunologically relevant lymphocyte subsets and 8 cytokines and important biomarkers were analysed at all time points.

**Results:**

Patients displayed no signs of discomfort or dose-related toxicities in response to MSC infusion. Flow cytometric analysis revealed an increase in B regulatory lymphocyte populations and nonconventional T regulatory cells and a decrease in T effector lymphocyte proportions in auto-MSC-infused patients. No such favourable immune responses were observed in all MSC-infused patients.

**Conclusion:**

This study provides evidence that auto-MSCs are safe and well tolerated. This is the first ever report to compare autologous and allogeneic MSC infusion in KTx patients. Importantly, our data demonstrated that MSC-induced immune responses in patients did not completely correlate with clinical outcomes. Our findings add to the current perspective of using MSCs in KTx and explore possibilities through which donor/recipient chimerism can be achieved to induce immune tolerance in KTx patients.

## 1. Introduction

Kidney transplantation (KTx) coupled with immunosuppressive (IS) drugs is the preferred treatment for end-stage renal disease (ESRD) [1] over the dialysis process, which is usually performed in case of nonavailability of a suitable donor or underlying medical conditions. Despite medical advances, long-term graft survival post-KTx continues to be a major challenge [2] further jeopardized by prolonged usage of IS drugs. Therefore, it would be of immense benefit to seek novel therapies that can replace/taper down the usage of immunosuppressants. The principal goal of IS therapy is immune cell inhibition [3]; however, exploiting these therapies against specific lymphocytes is difficult due to the existence of overlapping pathways used by effector and regulatory lymphocytes [4]. Therefore, it is essential to understand the effect of various cell-based therapies on lymphocyte compartments, as immunoregulatory mechanisms mediate the majority of posttransplant effects. In this regard, mesenchymal stromal cells (MSCs) have been shown to hold an immense potential to be considered an alternative or adjunct treatment for many diseased conditions for their potential of immunomodulation and regeneration through paracrine and direct effects, respectively. MSCs are well documented to affect T cells [5], but their effect has not been fully extrapolated on the T cell subset interplay. Recent studies have also highlighted the capacity of MSCs to modulate B cells [6, 7]. It would, therefore, be interesting to explore if MSCs reshape the immune balance in KTx patients and their effect on the graft outcome.

Our previously published pilot study in 4 KTx patients showed the safety and efficacy of auto-MSCs in combination with IS drug therapy postinduction [8]. The current study was designed to evaluate the effect of 2-time point MSC infusion on T and B cells in autologous and allogeneic (donor-derived) settings without any ATG induction therapy. And thereafter, the effect is analysed in the clinical setting.

## 2. Methods

### 2.1. Study Objectives, Design, Safety, and Efficacy Monitoring

Recruited patients were histopathologically confirmed for ESRD. Patients who received ATG induction therapy or were suffering from any infections were excluded. Inclusion and exclusion criteria are detailed in Supplementary Table (ST 1). All protocols designed were approved by the Institutional Committee for Stem Cell Research of PGIMER (PGI-IC-SCRT-39-2013/1471), Chandigarh, and no changes were made following approval. Protocols were performed according to relevant regulations and guidelines specified in the approval letter, and informed consent was obtained from the patients.

**Figure 1:**
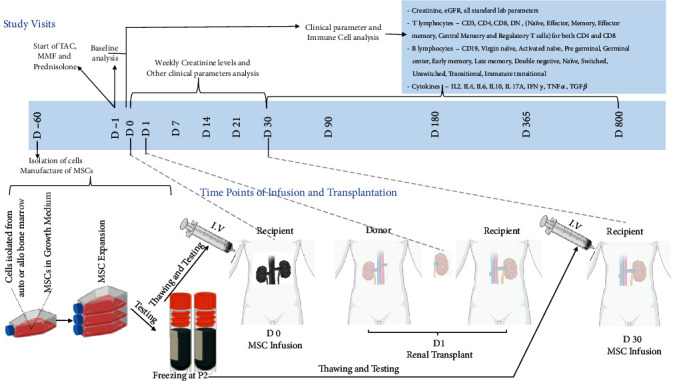
Treatment scheme for KTx patients recruited for the study. MSCs were isolated from bone marrow aspirate and expanded for 60 days (D-60) before transplantation. All KTx patients received immunosuppressive drugs (tacrolimus (TAC), mycophenolate mofetil (MMF), and prednisolone) 48 hours before transplantation (D-1). Allo and auto group patients received the 1^st^ intravenous (I.V.) MSC infusion 24 hours before transplantation (D-0) and the 2^nd^ I.V. MSC infusion 30 days posttransplantation (D-30). Blood samples were collected at D-0, D-30, D-90, D-180, D-365, and D-800 for determining clinical and immunological parameters. Samples were routinely processed for serum creatinine estimation.

For this open-label, parallel-group prospective study (Supplementary Figure (SF 1), a total of 30 patients, with planned KTx from a living-related donor, were assessed and 17 patients meeting the inclusion criteria were divided into 3 groups (SF 1), i.e., autologous (auto) group (*n* = 6; median age 24 (23, 27)), allogeneic (allo) group (*n* = 6; median age 31 (20.5, 37)), and control group (*n* = 5; median age 23.5 (25, 35)). The allocation ratio for the assignment was 1 : 1 : 1. Patients were enrolled from June 2013 till March 2015 and were followed up for 2 years. The primary and secondary objectives and endpoints have been summarized in ST 2. The patient demographics and clinical profile are described in ST 3, and the cell dosage is described in ST 4. One patient from each auto and allo group did not follow up, leaving *n* = 5 for each group. The study design involving different time points is graphically described in Figure 1. Lymphocyte population sets, cytokines, and biomarkers characterized are tabulated in Table 1. A statistical summary of all clinical and immunological parameters measured periodically is reported in ST 5 and Table 2, respectively.

### 2.2. Immunosuppressive Drug Treatment and Supportive Care

All patients received treatment with tacrolimus (TAC), mycophenolate mofetil (MMF), and prednisolone (Figure 1). TAC was started 48 hours before transplant surgery and adjusted to maintain a trough level of 10-12 ng/mL for the first month posttransplant and then between 8 and 10 ng/mL for the next 1-3 months. MMF (Cellcept®, Roche) at a dose of 1 g, twice a day, was given. Steroids at a dose of 0.5 mg/kg were given initially and tapered to 5 mg/day by the 6^th^ week posttransplant. Additionally, cotrimoxazole (400 mg sulfamethoxazole+160 mg trimethoprim) daily for 6 months was given. Whole blood TAC trough level (C0) was monitored till the target level was attained. Patients were monitored for changes in clinical condition or serum creatinine (Scr) levels.

The estimated glomerular filtration rate (eGFR) was determined using the Nankivell equation [9]. Biopsies were performed for allograft dysfunction or proteinuria.

### 2.3. Cell Preparation and Characterization

MSCs were prepared from bone marrow (BM) aspirate of patients (auto group) or the respective kidney donors (allo group) approximately 6-8 weeks (Figure 1) before transplantation in the Department of Translational and Regenerative Medicine, PGIMER, Chandigarh, as described previously [8, 10]. Briefly, 40 mL of bone marrow sample was subjected to density gradient centrifugation, and mononuclear cells were collected and resuspended in complete media (*α*-minimal essential media+10% pooled human platelet lysate). Cells were inoculated in T-225 flasks at a density of 0.3 − 0.4 × 10^6^ cells/cm^2^ and kept in an incubator with 5% CO_2_ at 37°C. MSCs were trypsinized at 80% confluency, expanded in hyperflasks till passage 2, and cryopreserved in liquid nitrogen till the time of infusion. Cryopreserved MSCs were revived and expanded in complete MEM containing 10% pHPL 7 days before infusion. On the day of infusion (D-0 or D-30), cells were trypsinized, and their count was determined using trypan blue (>95% viability) before infusion.

MSCs were also characterized phenotypically and functionally in accordance with International Society for Cell Therapy (ISCT) guidelines [11]. When observed under a light microscope, MSCs had typical spindle-shaped morphology and adhered to the surface (SF 2A). For phenotypic characterization, MSCs were stained with fluorochrome-labelled antibodies and were analysed using flow cytometry. Culture-expanded MSCs at passage 3 showed ≤2% immunoreactivity for haematopoietic lineage markers CD34, CD45, CD11b, CD19, and HLA-DR and ≥95% positivity for human-MSC specific markers CD73, CD90, and CD105 (SF 2B). Unstained MSCs were used as a negative control for analysis.

Functional characterization of MSCs was done at passage 4 based on their differentiation into adipocytes, osteocytes, and chondrocytes (SF 2C). For adipogenic differentiation, cells were plated in a 6-well plate at a density of 15 × 10^3^ cells/cm^2^ and maintained in an adipogenic medium comprising *α*-MEM, isomethylbutylxanthine, insulin, dexamethasone, and indomethacin. Similarly, for osteogenic differentiation, cells were plated at a density of 15 × 10^3^ cells/cm^2^ and with *α*-MEM supplemented with dexamethasone, ascorbic acid, and glycerophosphate. To evaluate chondrogenic potential, a chondrocyte differentiating kit (HiMedia) was used per the manufacturer's protocol.

The culture medium was changed every 3 to 4 days. On the 21st day, staining was performed using Oil Red O to estimate the neutral lipid accumulation in fat vacuoles of differentiated adipocytes. Likewise, the staining for differentiated osteocytes was performed using Alizarin Red S, which detects the alkaline phosphatase activity, and chondrogenic differentiation was demonstrated by staining with Alcian Blue, which detects the expression of aggrecans in chondrocytes.

Karyotyping was also performed for the culture-expanded MSCs (passage 3) to confirm chromosomal stability (SF 2D). By actively dividing cells from 70%, confluent culture flasks were treated with KaryoMAX® (Gibco) to inhibit the proliferation of cells at the metaphase stage. After the mitotic arrest, the cells were harvested using trypsin/EDTA and immersed in KCl solution at 37°C for hypotonic treatment. The treated cells were centrifuged, followed by fixation using Carnoy's fixative. Cells were resuspended in a fresh fixative solution at room temperature for slide preparation. The cell suspension was dropped on the slide and kept on a hot plate for 2-3 min at 38-40°C. Once dried, the slides were kept at room temperature overnight and afterwards were immersed in cold trypsin solution, and staining was performed using Giemsa. The trypsin and Giemsa bands (GTG) were analysed microscopically (100x). Metaphases were captured through a CCD camera and analysed using the GenASIs Bandview software (Applied Spectral Imaging). A minimum of 20 banded metaphases was captured for all samples.

MSC culture medium was used to detect bacterial and fungal contaminants or the incidence of mycoplasma pathogen (SF 2E). The BACTEC blood culture system was used to rule out aerobic and anaerobic bacterial contaminations, and agar plates were used to detect fungal contaminations. For mycoplasma testing, nested PCR using a mycoplasma detection set (TaKaRa) was performed. Cells were infused once the sterility was confirmed.

### 2.4. Cell Administration

Auto and allo group patients received two intravenous MSC infusions at D-0 and D-30, and for each dose, approximately 1-1.5 × 10^6^ cells/kg body weight were given (ST 4). Patients were premedicated with paracetamol and chlorpheniramine as a precautionary measure to prevent any reactions postinfusion. The patient's vitals were monitored for 4-6 hours postinfusion.

### 2.5. Clinical Evaluation

Routine clinical parameters (ST 5) were measured at days (D) 0, 30, 90, 180, 365, and 800, along with serum creatinine and eGFR.

### 2.6. Immunological Evaluation

Immunophenotyping was performed on isolated peripheral blood mononuclear cells, and cytokine assays were performed on serum samples collected at days (D) 0, 30, 90, 180, 365, and 800. Lymphocyte subpopulations were analysed using fluorochrome-labelled monoclonal antibodies on a FACSAria flow cytometer (ST 6). Th1/Th2/Th17 cytokines and TGF-*β*1 were quantified using commercially available kits (ST 6). Gating strategies for phenotyping are provided in Supplementary Figures SF 3 and 4.

### 2.7. Statistical Analysis

Analysis was undertaken by using in-house R scripts [12]. Wherever applicable, values were first adjusted to the respective parent population. Adjusted values were further normalized (min-max) before statistical analysis. Linear mixed models using the R package lme4, followed by ANOVA, were used to access significantly contributing factors. The R method Wilcox test - Wilcox.test(x, y, paired = FALSE) was used to carry out the Wilcoxon rank sum test in order to test the null hypothesis that the distributions of two variables under investigation differ by a location shift of mu = 0. Plots of the resultant values were created using the R method boxplot().

## 3. Results

### 3.1. MSCs are Well Tolerated with No Clinical Impact on Graft Survival

**Figure 2:**
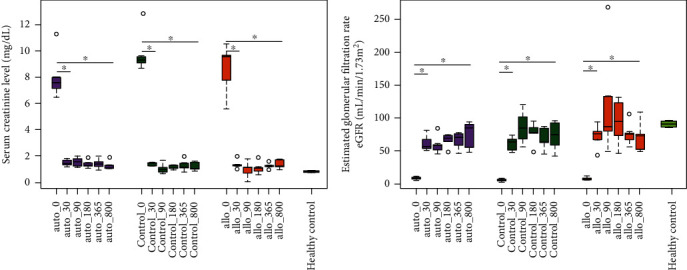
Distribution of kidney function biomarkers in kidney transplant patients. Quantification plots for (a) serum creatinine level (mg/dL) and (b) estimated glomerular filtration rate (eGFR) (mL/min/1.73 m^2^) at D-0, D-30, D-90, D-180, D-365, and D-800 time points for different groups (auto (*n* = 5), control (*n* = 5), allo (*n* = 5), and healthy control (*n* = 2)). Box plots show median of respective biomarker concentration. Significant differences are indicated as ^∗^*p* value < 0.05.

Patients displayed no signs of discomfort, allergies, or infections during or post-MSC infusion. In the auto group, 40% patients had rejection episodes immediately after transplantation (Pa5 and Pa6), and in the allo group as well, 40% patients had rejection episodes (P3—TCMR at 3.5 months, P6—immediate rejection posttransplantation) (ST 3), but afterwards stable graft function was achieved for all. On the contrary, no rejection episodes were observed in the control group (ST 3). All the routine clinical parameters analysed showed no significant changes over the period of follow-up and were in the normal range (ST 5). However, levels of Scr and eGFR are normalized within all groups posttransplantation (Figure 2).

### 3.2. MSCs Alter the Frequency of T and B Lymphocytes

**Figure 3:**
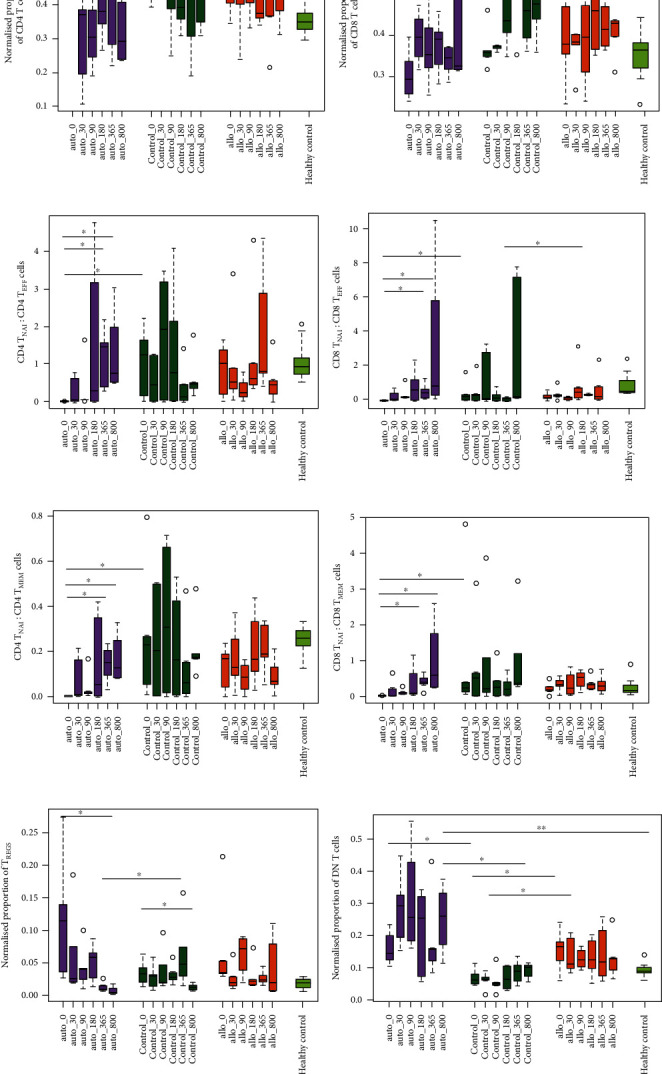
Lymphocyte subsets in peripheral blood of kidney transplant patients. Multicolour FACS analysis for the normalized proportion of (a) CD4 T cells and (b) CD8 T cells, (c) CD4 T_NAI_:CD4 T_EFF_ cells, (d) CD8 T_NAI_:CD8 T_EFF_ cells, (e) CD4 T_NAI_:CD4 T_MEM_ cells, (f) CD8 T_NAI_:CD8 T_MEM_ cells, (g) T_REGS_, and (h) DN T cells at D-0, D-30, D-90, D-180, D-365, and D-800 for different groups (auto (*n* = 5), control (*n* = 5), allo (*n* = 5), and healthy control (*n* = 3)). Box plots depict median of respective lymphocyte subsets. Significant differences are indicated as ^∗^*p* value < 0.05.

Flow cytometric analysis pointed that the auto group had a reduction in CD4 T cells at the end of follow-up while CD8 T cells remained unaffected (Figures 3(a) and 3(b)). We further compared metabolically inactive T_NAI_ cells to identify the impact of MSC infusion on the cell differentiation process.

Analysis of T_NAI_ against T_EFF_ cells revealed a higher proportion of T_NAI_ cells within the auto group at D-800 for both CD4 (Figure 3(c)) and CD8 T cells (Figure 3(d)). Additionally, analysis of T_NAI_:T_MEM_ cells showed elevation at D-800 within the auto group for both CD4 (Figure 3(e)) and CD8 (Figure 3(f)) T cell subsets. This points towards expanded T_NAI_ cells as compared to T_EFF_ and T_MEM_ cells in the auto group. This trend was more pronounced for the effector memory (T_MEM-EM_) subset than for the central memory (T_MEM-CM_) subset (Table 2). We found a drop in T_REGS_ in the auto and control groups and a decreasing trend in the allo group (Figure 3(g)). An increase in double-negative (DN) T cells at multiple time points was observed within the auto group, which turned out to be significantly higher at D-800 than that within the control group or healthy controls (Figure 3(h)). No significant differences in other T cell subsets were evident in either the allo or control group (Table 2).

In our previous study, CD19 B cells decreased in auto-MSC-treated patients posttransplantation [6]. To evaluate the specific impact of this change, we further characterized CD19 B cell subsets using bm-bm5 and CD27/IgD classification. No relevant difference was evident in either of the subsets within the auto or control group. Intriguingly, within the allo group, bm2, bm2′, and bm3+4 cells displayed a decrease at D-800 (Table 2). These results indicate differences in long-term effects of auto- and allo-MSCs on the B cell profile of KTx patients.

**Figure 4:**
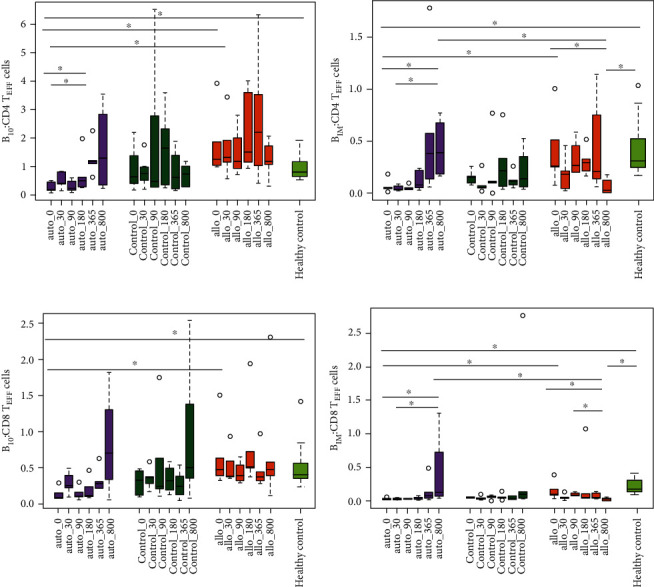
Comparison of regulatory B and effector T cell subset distribution in peripheral blood of kidney transplant patients. Multicolour FACS analysis for the normalized proportion of (a) B_10_:CD4 T_EFF_ cells, (b) B_IM_: CD4 T_EFF_ cells, (c) B_10_:CD8 T_EFF_ cells, and (d) B_IM_:CD8 T_EFF_ cells at D-0, D-30, D-90, D-180, D-365, and D-800 for different groups (auto (*n* = 5), control (*n* = 5), allo (*n* = 5), and healthy control (*n* = 3)). Box plots depict the median of the respective cell subset ratios. Significant differences are indicated as ^∗^*p* value < 0.1 and ^∗∗^*p* value < 0.05.

Since B_REGS_ contribute to transplant tolerance, we studied immature B (B_IM_) cells along with other two B_REG_ subsets, with phenotypes similar to classical B_reg_ and B_10_ populations, i.e., CD19^+^CD5^+^CD1d^hi^ (B_regs_) and CD19^+^CD27^+^CD24^hi^ (B_10_ cells). Comparative analysis of B_10_ and B_IM_ subsets against CD4/CD8 T_EFF_ populations indicated a significant increase within the auto group at varied time points (Figures 4(a)–4(d)).

On the contrary, in the allo group, B_IM_:T_EFF_ cells reduced within the group and variedly decreased when compared to the HC (Table 2). No significant changes were observed in B cell populations within the control group.

### 3.3. MSCs Modulate Cytokine Levels

A significant increase in TGF-*β*1 levels was evident within the auto group until D-800 (Table 2), which overlapped with a decrease in CD4 T_EFF_ cells and an increase in DN T and B_REG_ subsets. The allo group showed an intermittent increase in TGF-*β*1 post-D-30 (Table 2). IL-2 MFI in the auto group at D-365 was lower than that in the allo group (Table 2). None of the other cytokines had any significant changes in the auto or allo group (Table 2). No significant differences in cytokine levels were observed in the control group.

## 4. Discussion

Immunosuppressants are given to KTx recipients to hamper immune cell-mediated rejection, thereby promoting successful engraftment of the donor kidney. Despite improvements in IS drug management, KTx patients not only suffer from life-threatening complications but are also predisposed to opportunistic infections. There is an utmost need to develop an approach that will aid donor-specific immune-hyporesponsiveness, thereby reducing the patient's dependence on immunosuppressants. The immune milieu is ever changing, and graft acceptance in transplant settings is determined by how well the immune system adapts to challenges that an engraft imposes. Nevertheless, simultaneous assessment of the cellular and humoral arm of the immune system is paramount in the transplant setting for predicting graft quality.

MSCs have been major contenders for their potential towards therapeutic, regenerative, and immunomodulatory activities. This study evaluates safety and efficacy of auto-MSCs and allo-MSCs in patients who underwent KTx. The first infusion was given at D-0 to establish a protolerogenic microenvironment that might promote graft acceptance and avoid acute deterioration of graft function, and the second infusion at D-30 was given to combat inflammatory environment postsurgery and to prolong protolerogenic effect mediated by MSCs.

Our study signifies that MSC infusion is feasible with favourable immune response in renal transplant patients, but there is no short-term clinical benefit of such an intervention in a normal risk renal transplant. We show that auto-MSC infusion upregulates naive T (T_NAI_) subsets, and B regulatory (B_REGS_) and double-negative (DN) T cells may contribute to a decrease in circulating effector T (T_EFF_) cells.

It is known that donor-specific tolerance is considered Holy Grail for transplant immunology, and studies suggest that T_MEM_ cells can directly stimulate T_EFF_ cells and prove to be deleterious to the graft [13]. There has been increased incidence of rejections related to increased circulating memory T cells [14]. Therefore, low T_EFF_/T_MEM_ cell proportions relative to T_NAI_ cells post-auto-MSC infusion that were observed in our study could be of potential therapeutic value.

T_REGS_ have been reported to maintain donor-specific nonresponsiveness in KTx patients [15]; however, we found a drop in T_REGS_ in all groups irrespective of MSC infusion, which challenges the present view of MSC-induced T_REGS_ expansion. However, the number of T_REGS_ might not even correlate with the functional ability of MSCs to suppress T cell functions [16]. Downregulation of T_REGS_ can be attributed to the use of calcineurin inhibitors as a part of IS therapy [17], which is known to block IL-2 production, required for T_REGS_ expansion. Lesser-known nonconventional T_REGS_ subsets such as double-negative T (DN T) cells are also known to have immunosuppressive properties [18]. DN T cells lack FoxP3 expression and therefore are resistant to calcium release-activated calcium channel inhibition [19] which supports the increase in these cells in our study. Also, studies so far have suggested the importance of B_REGS_ in preclinical transplant models and patients [20, 21]. B_REGS_ have a direct impact by inhibiting effector T cells in addition to their role in antibody production. Their profiling may help identify patients with immunotolerance thereby minimizing immunosuppressive regimens. The increasing trend of B_REGS_ in relation to effector T cells in our setup indicates well-guarded B cell tolerance checkpoints post-auto-MSC infusion; however, the functional status of these cells has not been determined in our study.

On the contrary, allo-MSC infusion led to no significant change in T cell subsets but decreased regulatory B cell subsets. Although various studies advocate the use of allo-MSCs (Table 3), our data suggest that prior to considering the application of MSCs of allo origin in kidney transplant patients, further studies are needed to analyse their effects on the immune cell phenotype and function.

We identified TGF-*β* as the primary immunomodulatory cytokine in our study. Increase in this anti-inflammatory cytokine might indicate a shift from Th1 to Th2 response in auto group patients.

MSCs have been major contenders for their potential towards immunomodulatory properties [22, 23].

Numerous studies have reported the safety and efficacy of MSCs (Table 3); however, there are differences in the source, dosage, route of MSC administration, time points, IS regimen, and follow-up periods. Moreover, differences in efficacy endpoints of these studies make it further challenging to infer the therapeutic efficacy of MSCs.

**Figure 5:**
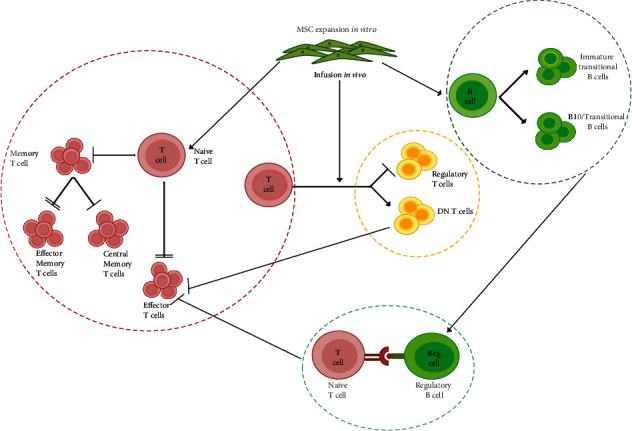
Model of immune cell regulation post-MSC administration in autologous KTx patients. MSC infusion leads to increase in naïve T cells, B_REGS_, and DN T cells and decrease in the memory and effector T cell population. Increase in B_REGS_ and DN T cell possibly inhibits T_EFF_ cell function. B_REGS_ can also act as antigen-presenting cells (APC) which form immune complexes with T_NAI_ cells ultimately leading to T_EFF_ cell apoptosis.

The novelty of our study lies in the comparison of the immune profile of two groups administered with 2 time point doses of autologous and allogeneic MSCs, and the major findings point towards a controlled immune environment (Figure 5) for the graft, especially in the auto group with lesser impact on clinical parameters used for determining the graft survivability. While few of the studies, as pointed in Table 3, have pointed towards basic immune repertoire, some have pointed towards clinical safety and feasibility of auto- or allo-MSCs. Our study is unique in comparative analysis of 29 T and B cell subsets with cytokine profiling in two groups with an uncertain impact on clinical outcome, emphasizing conducting more regulated trials utilizing MSCs in solid organ transplantation.

Our study is limited by small sample size and lack of functional assessment data. However, our findings would contribute substantially toward understanding the long-term immunomodulatory effects of MSCs, considering the inadequacy of available MSC efficacy data. Although we believe that favourable immune response is taking the front seat post-auto-MSC infusion, clinical relevance can only be stated upon in large sample size and more follow-up years.

The primary outcome (ST 2) of the study is that the infusion of auto-MSCs is safe and well tolerated in KTx patients. As far as the graft outcome is concerned, all KTx patients showed stable graft function eventually after rejection episodes in few patients. Variations in immunological responses were evident, regardless of the same origin, isolation, expansion conditions, and dosage of MSCs. The exact reason behind these differences remains unclear; however, these could have been elicited by donor-dependent variability or host microenvironment. As a secondary outcome (ST 2), the results collectively stress upon a unique trend of change in lymphocyte subsets that will help us to conduct more targeted clinical trials to improve long-term graft survival eventually. MSCs of autologous origin may be the safer choice in terms of avoiding unwanted immune responses while MSCs of allogenic origin might elicit specific cellular and humoral immune responses against donor antigens.

In spite of a seemingly favourable immune profile, the clinical ineffectiveness is evident in this study. Therefore, our findings add to the current perspective of using MSCs in KTx and explore possibilities through which donor/recipient chimerism can be achieved to induce immune tolerance in KTx patients.

## Figures and Tables

**Table 1:** Lymphocyte population sets, cytokines, and biomarkers characterized.

Lymphocyte populations		
Parent populations	Subsets	Phenotype
1. Mononuclear cells	1. CD3 lymphocytes (T cells)	CD3^+^
2. CD3^+^ T cells	2. CD4 lymphocytes (helper T cells)	CD3^+^CD4^+^
	3. CD8 lymphocytes (cytotoxic T cells)	CD3^+^CD8^+^
	4. Double-negative T cells (DN T)	CD3^+^CD4^−^CD8^−^
3. CD3^+^ CD4^+^ T cells/helper T cells	5. Naive T (T_NAI_) cells	CD3^+^CD4^+^CD45RA^+^CD45RO^−^CD62L^+^
	6. Effector T (T_EFF_) cells	CD3^+^CD4^+^CD45RA^+^CD45RO^−^CD62L^−^
	7. Memory T (T_MEM_) cells	CD3^+^CD4^+^CD45RA^−^CD45RO^+^
	8. Effector memory T (T_MEM-EM_) cells	CD3^+^CD4^+^CD45RA^−^CD45RO^+^CD62L^−^
	9. Central memory T (T_MEM-CM_) cells	CD3^+^CD4^+^CD45RA^−^CD45RO^+^CD62L^+^
	10. Regulatory T cells (T_REGS_)	CD3^+^CD4^+^CD25^hi^FoxP3^+^CD127^lo^
4. CD3^+^CD8^+^ T cells/cytotoxic T cells	11. Naive T (T_NAI_) cells	CD3^+^CD8^+^CD45RA^+^CD45RO^−^CD62L^+^
	12. Effector T (T_EFF_) cells	CD3^+^CD8^+^CD45RA^+^CD45RO^−^CD62L^−^
	13. Memory T (T_MEM_) cells	CD3^+^CD8^+^CD45RA^−^CD45RO^+^
	14. Effector memory T (T_MEM-EM_) cells	CD3^+^CD8^+^CD45RA^−^CD45RO^+^ CD62L^−^
	15. Central memory T (T_MEM-CM_) cells	CD3^+^CD8^+^CD45RA^−^CD45RO^+^ CD62L^+^
5. CD19^+^ B cells	16. Virgin naive (bm1) B cells	CD19^+^IgD^+^CD38^−^
	17. Activated naive (bm2) B cells	CD19^+^IgD^+^CD38^+^
	18. Pregerminal (bm2′) B cells	CD19^+^IgD^+^CD38^hi^
	19. Germinal centre (GC) (bm3+4) B cells	CD19^+^IgD^−^CD38^hi^
	20. Early memory (early bm5) B cells	CD19^+^IgD^−^CD38^+^
	21. Late memory (late bm5) B cells	CD19^+^IgD^−^CD38^−^
	22. Double-negative B (DN B) cells	CD19^+^IgD^−^CD27^−^
	23. Naive B cells	CD19^+^IgD^+^CD27^−^
	24. Switched B cells	CD19^+^IgD^−^CD27^+^
	25. Unswitched B cells	CD19^+^IgD^+^CD27^+^
6. CD19^+^ B regulatory cells (B_REGS_)	26. Regulatory B (B_reg_) cells	CD19^+^CD5^+^CD1d^hi^
	27. Transitional B (B_10_) cells	CD19^+^CD27^+^CD24^hi^
	28. Immature transitional B (B_IM_) cells	CD19^+^CD24^hi^CD38^hi^
Cytokines analysed		
1. Interleukin-2 (IL-2)		
2. Interleukin-4 (IL-4)		
3. Interleukin-6 (IL-6)		
4. Interleukin-10 (IL-10)		
5. Interleukin-17A (IL-17A)		
6. Interferon-*γ* (IFN-*γ*)		
7. Tumor necrosis factor-*α* (TNF-*α*)		
8. Transforming growth factor-*β*1 (TGF-*β*1)		
Biomarkers analysed		
1. Serum creatinine (Scr)		

**Table 2:** Statistical summary of immunological parameters analysed in different study groups.

Parameter	Group	Day 0 (baseline)	Day 30	Day 90	Day 180	Day 365	Day 800
CD3 proportion	Allo	0.46 (0.44, 0.49)	0.40 (0.36, 0.44)^∗^	0.49 (0.35, 0.63)	0.47 (0.27, 0.55)	0.35 (0.33, 0.46)	0.22 (0.16, 0.40)
	Auto	0.35 (0.22, 0.41)^!^	0.15 (0.02, 0.23)^∗^,^§^	0.11 (0.03, 0.20)^∗^,^§^	0.15 (0.09, 0.35)	0.22 (0.09, 0.38)	0.18 (0.09, 0.31)
	Control	0.40 (0.22, 0.47)	0.50 (0.36, 0.52)	0.42 (0.19, 0.50)	0.32 (0.18, 0.48)	0.50 (0.23, 0.55)	0.22 (0.20, 0.40)
	Healthy control	0.28 (0.13, 0.47)					
CD4 proportion	Allo	0.46 (0.37, 0.53)	0.52 (0.32, 0.58)	0.50 (0.37, 0.57)	0.37 (0.35, 0.51)	0.42 (0.29, 0.52)	0.44 (0.35, 0.51)
	Auto	0.51 (0.45, 0.59)	0.37 (0.15, 0.44)^∗^,§	0.30 (0.22, 0.40)^∗^,^!^	0.38 (0.30, 0.55)	0.48 (0.25, 0.55)	0.29 (0.24, 0.44)^∗^,^¶^
	Control	0.55 (0.44, 0.59)	0.55 (0.53, 0.56)	0.44 (0.32, 0.53)	0.39 (0.33, 0.42)^∗^	0.47 (0.25, 0.48)	0.42 (0.33, 0.46)
	Healthy control	0.48 (0.45, 0.52)					
CD8 proportion	Allo	0.38 (0.30, 0.48)	0.38 (0.32, 0.48)	0.40 (0.28, 0.50)	0.46 (0.36, 0.53)	0.47 (0.37, 0.52)	0.43 (0.35, 0.49)
	Auto	0.29 (0.25, 0.37)	0.39 (0.33, 0.46)	0.35 (0.29, 0.45)	0.39 (0.31, 0.43)	0.34 (0.30, 0.48)	0.33 (0.32, 0.57)
	Control	0.36 (0.33, 0.41)	0.37 (0.36, 0.38)	0.43 (0.38, 0.60)	0.50 (0.42, 0.54)	0.46 (0.38, 0.62)	0.48 (0.40, 0.57)
	Healthy control	0.44 (0.37, 0.46)					
CD4 T_NAI_:T_EFF_ cell proportion	Allo	0.30 (0.09, 1.51)	0.53 (0.19, 2.14)	0.23 (0.06, 0.63)	0.45 (0.39, 2.66)	0.80 (0.58, 3.44)	0.44 (0.10, 1.06)
	Auto	0.003 (0.001, 0.007)^§^	0.008 (0.001, 0.7)	0.05 (0.02, 0.84)	0.27 (0.001, 3.96)	1.47 (0.35, 1.86)^∗^	0.76 (0.50, 2.5)^∗^
	Control	1.25 (0.07, 1.92)	0.45 (0.0001, 1.24)	1.91 (0.008, 3.33)	0.76 (0.004, 3.11)	0.13 (0.01, 0.92)	0.47 (0.25, 1.13)
	Healthy control	0.88 (0.57, 1.31)					
CD8 T_NAI_:T_EFF_ cell proportion	Allo	0.18 (0.06, 0.51)	0.27 (0.15, 0.70)	0.18 (0.04, 0.27)	0.43 (0.13, 1.94)	0.31 (0.28, 0.42)§	0.24 (0.08, 1.59)
	Auto	0.01 (0.004, 0.02)^§^	0.07 (0.008, 0.59)	0.35 (0.15, 0.55)	0.05 (0.10, 1.74)	0.46 (0.13, 0.95)^∗^	0.85 (0.24, 8.12)^∗^
	Control	0.26 (0.03, 1.02)	0.34 (0.0005, 1.20)	0.10 (0.03, 3.04)	0.16 (0.003, 0.57)	0.10 (0.006, 0.20)	0.21 (0.15, 7.43)
	Healthy control	0.34 (0.22, 1.42)					
CD4 T_NAI_:T_MEM_ cell proportion	Allo	0.17 (0.02, 0.20)	0.13 (0.05, 0.31)	0.08 (0.01, 0.15)	0.16 (0.07, 0.38)	0.19 (0.11, 0.32)	0.06 (0.03, 0.17)
	Auto	0.0007 (0.0004, 0.002) §	0.001 (0.0006, 0.19)	0.01 (0.007, 0.09)^∗^	0.06 (0.0002, 0.38)	0.15 (0.06, 0.21)^∗^	0.13 (0.08, 0.29)^∗^^,^^
	Control	0.23 (0.03, 0.53)	0.20 (0.0001, 0.50)	0.30 (0.007, 0.69)	0.16 (0.007, 0.48)	0.06 (0.006, 0.31)	0.17 (0.12, 0.33)
	Healthy control	0.25 (0.20, 0.30)					
CD8 T_NAI_:T_MEM_ cell proportion	Allo	0.22 (0.08, 0.37)	0.31 (0.14, 0.49)	0.23 (0.03, 0.70)	0.50 (0.18, 0.70)	0.35 (0.21, 0.52)	0.27 (0.10, 0.57)
	Auto	0.01 (0.01, 0.01)^§^	0.03 (0.01, 0.43)	0.03 (0.02, 0.18)	0.06 (0.02, 0.88)	0.38 (0.21, 0.58)^∗^^,^^	0.61 (0.24, 2.17)^∗^^,^^
	Control	0.23 (0.08, 2.60)	0.51 (0.001, 1.91)	0.21 (0.05, 2.49)	0.25 (0.009, 0.80)	0.20 (0.01, 0.55)	0.35 (0.30, 2.20)
	Healthy control	0.20 (0.12, 0.44)					
CD4 T_NAI_: T_MEM-EM_ cells	Allo	0.22 (0.02, 0.38)^¶^	0.16 (0.06, 0.49)	0.19 (0.03, 0.20)	0.28 (0.10, 0.60)	0.32 (0.15, 0.58)^^^	0.08 (0.04, 0.24)
	Auto	0.0007 (0.0004, 0.002)^§,¶¶^	0.002 (0.0006, 0.235)	0.0162 (0.0078, 0.1466)	0.063 (0.0002-0.7132)	0.26 (0.08, 0.40)^∗^	0.20 (0.10, 0.43)^∗^
	Control	0.32 (0.03, 1.20)	0.27 (0.0001, 0.95)	0.48 (0.0074, 1.36)	0.25 (0.0019, 0.80)	0.07 (0.006, 0.49)	0.21 (0.15, 0.48)
	Healthy control	0.36 (0.27, 0.45)					
CD8 T_NAI_:T_MEM-EM_ cells	Allo	0.29 (0.08, 0.43)	0.42 (0.16, 0.54)	0.41 (0.50, 0.68)	0.69 (0.20, 0.80)	0.38 (0.26, 0.64)	0.37 (0.11, 0.64)
	Auto	0.012 (0.01, 0.014)^§,¶¶^	0.03 (0.01, 0.47)	0.04 (0.02, 0.19)	0.06 (0.02, 1.26)	0.42 (0.25, 0.65)^∗^	0.41 (0.31, 2.10)^∗^
	Control	0.26 (0.09, 3.39)	0.59 (0.001, 2.34)	0.23 (0.05, 2.93)	0.27 (0.01, 0.90)	0.21 (0.01, 0.60)	0.43 (0.33, 2.43)
	Healthy control	0.22 (0.15, 0.49)					
CD4 T_NAI_:T_MEM-CM_ cells	Allo	0.44 (0.10, 0.79)	0.60 (0.23, 0.95)	0.65 (0.14, 1.16)	0.40 (0.26, 1.18)	0.73 (0.32, 0.78)	0.42 (0.15, 0.63) ^§,¶^
	Auto	0.05 (0.02, 0.09)^§,¶¶^	0.08 (0.05, 0.94)	0.21 (0.07, 0.38)	0.43 (0.02, 0.86)	0.38 (0.26, 0.50)^∗^	0.65 (0.41, 0.71)^∗^^,§^
	Control	0.69 (0.44, 1.07)	0.82 (0.05, 1.07)	0.86 (0.31, 1.50)	0.45 (0.06, 1.20)	0.35 (0.11, 0.92)	0.99 (0.66, 1.32)
	Healthy control	0.79 (0.53, 0.95)					
CD8 T_NAI_:T_MEM-CM_ cells	Allo	1.3 (0.53, 3.42)	2.39 (0.91, 5.90)	3.00 (1.13, 4.65)	3.70 (1.44, 5.28)	2.30 (1.09, 4.72)	1.47 (0.67, 7.38)
	Auto	0.53 (0.37, 1.83)^¶^	1.29 (0.59, 7.17)	2.67 (0.55, 2.72)	1.71 (0.79, 6.9)	4.59 (1.54, 5.13)	3 (1.6, 11.48)^∗^
	Control	1.97 (1.44, 11.47)	4 (0.12, 11.25)	3.85 (1.01, 17.42)	2.49 (0.16, 7.83)	3.65 (0.39, 7.49)	2.63 (2.23, 35.81)
	Healthy control	2.33 (1.56, 5.32)					
T_REGS_ proportion	Allo	0.03 (0.03, 0.13)	0.02 (0.01, 0.05)	0.07 (0.03, 0.09)	0.02 (0.02, 0.05)	0.02 (0.02, 0.04)	0.02 (0.01, 0.09)
	Auto	0.11 (0.03, 0.21)^¶^	0.02 (0.02, 0.13)	0.04 (0.01, 0.07)	0.06 (0.02, 0.07)	0.01 (0.01, 0.01)^∗^^,§^	0.004 (0.002, 0.01)^∗^^,#,^^
	Control	0.03 (0.02, 0.05)	0.03 (0.01, 0.04)	0.02 (0.02, 0.07)	0.03 (0.02, 0.04)	0.05 (0.02, 0.11)	0.01 (0.01, 0.02)^∗^
	Healthy control	0.02 (0.01, 0.03)					
DN T cell proportion	Allo	0.17 (0.09, 0.21)^§^	0.11 (0.09, 0.20)^§^	0.12 (0.10, 0.16)	0.13 (0.08, 0.19)	0.12 (0.07, 0.24)	0.13 (0.08, 0.19)
	Auto	0.14 (0.11, 0.22)^§^	0.29 (0.18, 0.39)^§^	0.26 (0.17, 0.49)^!,§^	0.25 (0.06, 0.33)	0.16 (0.10, 0.30)	0.26 (0.14, 0.35)^§,¶¶^
	Control	0.05 (0.05, 0.10)	0.07 (0.04, 0.08)	0.05 (0.03, 0.09)	0.07 (0.03, 0.10)	0.09 (0.05, 0.12)	0.10 (0.07, 0.11)
	Healthy control	0.09 (0.08, 0.11)					
Virgin naive B (bm1) cell proportion	Allo	0.10 (0.07, 0.44)	0.13 (0.09, 0.49)	0.19 (0.07, 0.43)	0.12 (0.10, 0.42)	0.12 (0.09, 0.45)	0.26 (0.12, 0.52)
	Auto	0.14 (0.08, 0.27)	0.15 (0.10, 0.20)	0.15 (0.08, 0.18)	0.14 (0.06, 0.21)	0.21 (0.07, 0.13)	0.11 (0.08, 0.30)
	Control	0.16 (0.13, 0.18)	0.18 (0.13, 0.26)	0.15 (0.06, 0.21)	0.17 (0.12, 0.23)	0.15 (0.12, 0.19)	0.17 (0.07, 0.26)
	Healthy control	0.14 (0.09, 0.27)					
Activated naive (bm2) cell proportion	Allo	0.25 (0.15, 0.25)^¶^	0.30 (0.11, 0.33)	0.25 (0.14, 0.29)	0.25 (0.16, 0.30)	0.28 (0.14, 0.34)	0.04 (0.01, 0.15)^∗^^,#^
	Auto	0.28 (0.14, 0.36)	0.25 (0.11, 0.41)	0.35 (0.16, 0.42)	0.26 (0.15, 0.39)	0.29 (0.15, 0.36)	0.16 (0.09, 0.27)^¶^
	Control	0.32 (0.24, 0.39)	0.33 (0.23, 0.40)	0.40 (0.17, 0.41)	0.31 (0.21, 0.34)	0.32 (0.18, 0.39)	0.17 (0.08, 0.26)^∗^^,¶^
	Healthy control	0.31 (0.25, 0.38)					
Pregerminal (bm2′) cell proportion	Allo	0.17 (0.05, 0.29)	0.10 (0.05, 0.19)	0.19 (0.08, 0.26)	0.12 (0.05, 0.17)	0.15 (0.04, 0.20)	0.005 (0.002, 0.060)^^,¶^
	Auto	0.04 (0.01, 0.23)	0.03 (0.02, 0.15)	0.11 (0.03, 0.21)	0.05 (0.03, 0.21)	0.12 (0.70, 0.31)	0.11 (0.04, 0.14)
	Control	0.20 (0.12, 0.21)	0.15 (0.09, 0.20)	0.19 (0.05, 0.22)	0.11 (0.07, 0.24)	0.20 (0.11, 0.22)	0.06 (0.05, 0.31)
	Healthy control	0.19 (0.05, 0.33)					
Germinal center- GC (bm3+4) cell proportion	Allo	0.05 (0.03, 0.09)	0.03 (0.01, 0.03)	0.05 (0.03, 0.08)	0.07 (0.02, 0.13)	0.06 (0.03, 0.09)^#^	0.01 (0.001, 0.02)^∗^^,^,§,¶^
	Auto	0.02 (0.01, 0.04)	0.02 (0.008, 0.39)	0.013 (0.008, 0.04)	0.02 (0.004, 0.10)	0.02 (0.008, 0.09)	0.05 (0.03, 0.09)^!^
	Control	0.05 (0.03, 0.10)	0.03 (0.02, 0.05)	0.02 (0.01, 0.26)	0.07 (0.03, 0.14)	0.04 (0.02, 0.13)	0.05 (0.02, 0.09)
	Healthy control	0.02 (0.02, 0.04)					
DN B cells	Allo	0.05 (0.02, 0.08)^§^	0.07 (0.03, 0.14)	0.07 (0.02, 0.10)	0.05 (0.03, 0.08)	0.06 (0.02, 0.14)	0.15 (0.08, 0.34)
	Auto	0.18 (0.12, 0.35)!	0.21 (0.11, 0.34)!	0.18 (0.12, 0.29)!	0.14 (0.12, 0.28)!	0.15 (0.12, 0.25)	0.10 (0.08, 0.15)
	Control	0.13 (0.12, 0.23)	0.16 (0.11, 0.21)	0.09 (0.06, 0.26)	0.22 (0.08, 0.20)	0.12 (0.08, 0.17)	0.13 (0.08, 0.24)
	Healthy control	0.12 (0.03, 0.16)					
B_NAI_ cell proportion	Allo	0.53 (0.30, 0.59)	0.49 (0.36, 0.62)	0.47 (0.37, 0.57)	0.59 (0.29, 0.60)	0.51 (0.41, 0.65)	0.45 (0.26, 0.54)
	Auto	0.44 (0.40, 0.54)^¶^	0.40 (0.25, 0.50)	0.52 (0.31, 0.63)	0.43 (0.26, 0.60)	0.56 (0.28, 0.60)	0.53 (0.45, 0.63)
	Control	0.42 (0.20, 0.63)	0.42 (0.19, 0.59)	0.41 (0.09, 0.63)	0.31 (0.21, 0.49)	0.29 (0.17, 0.62)	0.41 (0.21, 0.64)
	Healthy control	0.67 (0.54, 0.82)					
B_regs_:CD4 effector cells	Allo	0.06 (0.03, 0.38)	0.02 (0.01, 0.18)	0.06 (0.04, 0.29)	0.06 (0.03, 0.20)	0.08 (0.03, 0.19)	0.01 (0.003, 0.03)
	Auto	0.02 (0.003, 0.03)	0.02 (0.01, 0.04)	0.01 (0.01, 0.02)	0.11 (0.02, 0.13)	0.05 (0.02, 0.43)	0.07 (0.02, 0.08)^!^
	Control	0.04 (0.03, 0.05)	0.03 (0.01, 0.06)	0.05 (0.02, 0.09)	0.07 (0.06, 0.30)^∗^	0.07 (0.02, 0.10)	0.03 (0.02, 0.05)
	Healthy control	0.02 (0.01, 0.04)					
B_regs_:CD8 effector cells	Allo	0.02 (0.01, 0.14)	0.01 (0.002, 0.06)	0.03 (0.01, 0.07)	0.01 (0.01, 0.083)	0.01 (0.01, 0.03)	0.002 (0.002, 0.01)^§^
	Auto	0.01 (0.002, 0.01)	0.01 (0.003, 0.02)	0.005 (0.004, 0.01)	0.03 (0.005, 0.04)	0.01 (0.01, 0.11)	0.03 (0.005, 0.122)
	Control	0.01 (0.01, 0.02)	0.01 (0.01, 0.03)	0.02 (0.01, 0.03)	0.02 (0.02, 0.07)	0.02 (0.01, 0.03)	0.02(0.01, 0.15)
	Healthy control	0.01 (0.003, 0.03)					
B_10_:CD4 effector cells	Allo	1.25 (1.01, 2.90)	1.33 (0.86, 2.68)	1.18 (0.83, 2.41)	1.51 (1.04, 3.81)	2.20 (0.72, 4.93)	1.19 (0.68, 1.90)
	Auto	0.18 (0.12, 0.49)^!,¶^	0.42 (027, 0.82)^!^	0.23 (0.13, 0.55)^!^	0.51 (0.27, 1.30)	1.17 (0.86, 1.7)^∗^^,#, ^^	1.30 (0.29, 3.19)
	Control	0.63 (0.29, 1.79)	0.75 (0.36, 1.37)	0.48 (0.28, 4.65)	1.64 (0.30, 2.96)	0.63 (0.17, 1.64)	0.74 (0.27, 1.10)
	Healthy control	0.68 (0.42, 1.25)					
B_10_:CD8 effector cells	Allo	0.47 (0.35, 1.06)	0.38 (0.36, 0.76)	0.39 (0.30, 0.59)	0.50 (0.43, 1.33)	0.37 (0.30, 0.70)	0.47 (0.25, 1.44)
	Auto	0.08 (0.07, 0.22)^!,¶^	0.25 (0.15, 0.44)	0.10 (0.07, 0.23)	0.11 (0.09, 0.35)	0.28 (0.21, 0.46)	0.70 (0.19, 1.56)
	Control	0.33 (0.10, 0.47)	0.37 (0.22, 0.48)	0.24 (0.15, 1.19)	0.31 (0.15, 0.54)	0.23 (0.08, 0.46)	0.50 (0.21, 1.96)
	Healthy control	0.38 (0.28, 0.62)					
B_IM_:CD4 effector cells	Allo	0.26 (0.17, 0.77)	0.18 (0.04, 0.34)	0.27 (0.20, 0.53)	0.30 (0.19, 0.43)	0.21 (0.10, 0.95)	0.03 (0.01, 0.16)^∗^^,^,¶^
	Auto	0.05 (0.03, 0.12)^!,¶^	0.04 (0.03, 0.08)	0.05 (0.04, 0.08)	0.08 (0.04, 0.23)	0.38 (0.10, 1.18)^∗^^,#,^^	0.39 (0.18, 0.73)^∗^^,#,^,!^
	Control	0.15 (0.09, 0.21)	0.06 (0.04, 0.17)	0.11 (0.05, 0.45)	0.22 (0.05, 0.55)	0.12 (0.07, 0.19)	0.14 (0.05, 0.44)
	Healthy control	0.27 (0.17, 0.61)					
B_IM_:CD8 effector cells	Allo	0.09 (0.06, 0.28)	0.05 (0.02, 0.10)	0.09 (0.08, 0.13)	0.05 (0.04, 0.59)	0.05 (0.04, 0.13)	0.03 (0.003, 0.05)^∗^^,^,¶^
	Auto	0.03 (0.02, 0.05)^!,¶^	0.03 (0.01, 0.04)	0.02 (0.02, 0.03)^§^	0.02 (0.01, 0.07)	0.07 (0.03, 0.31)	0.12 (0.06, 1.02)^∗^^,#,^,!^
	Control	0.05 (0.04, 0.06)	0.04 (0.02, 0.07)	0.06 (0.03, 0.08)	0.05 (0.03, 0.10)	0.03 (0.02, 0.07)	0.10 (0.04, 1.45)
	Healthy control	0.16 (0.09, 0.36)					
IL-2	Allo	136.70 (127.90, 146.20)^§^	129.80 (124.60, 154.80)	132.00 (128.40, 135.60)	126.60 (124.70, 136.10)	141.80 (133.60, 156.90)	ND
	Auto	126.00 (119.70, 139.90)	127.30 (123.20, 132.30)	132.30 (124.10, 139.50)	130.40 (124.10, 133.20)	121.60 (117.20, 131.70)^!!^	ND
	Control	119.70 (116.20, 124.10)	124.70 (74.66, 134.20)	120.30 (117.50, 126.60)	127.30 (109.60, 152.10)	122.20 (118.40, 161.30)	ND
TNF-*α*	Allo	107.10 (102.10, 115.30)	107.70 (104.10, 126.90)^§^	106.20 (98.12, 109.00)	101.40 (100.50, 108.00)^§^	104.60 (104.30, 112.50)	ND
	Auto	98.91 (92.61, 107.40)	96.39 (95.13, 100.80)^!^	97.65 (96.39, 105.80)	92.61 (91.04, 104.3)	97.02 (88.2, 103.60)	ND
	Control	91.98 (87.57, 99.23)	92.61 (18.90, 96.71)	90.72 (80.33, 98.91)	89.46 (80.33, 91.67)	96.39 (89.46, 102.70)	ND
IFN-*γ*	Allo	101.40 (99.54, 104.00)	90.09 (85.05, 97.97)	104.90 (98.75, 109.60)^#,§^	98.91 (93.24, 101.10)^§^	100.8 (93.87, 105.20)	ND
	Auto	107.10 (88.20, 124.70)	93.24 (87.89, 94.82)	98.91 (87.88, 138.00)	95.76 (86.63, 103.00)	90.09 (87.57, 97.02)	ND
	Control	91.98 (90.09, 101.70)	97.02 (89.78, 115.30)	85.05 (73.40, 91.35)	84.42 (80.96, 96.08)	86.94 (77.49, 98.60)	ND
IL-17A	Allo	73.08 (72.45, 84.42)	75.6 (66.62, 88.83)	81.27 (74.97, 93.24)	78.12 (71.19, 86.94)	85.68 (78.75, 99.23)^∗^	ND
	Auto	78.75 (68.67, 95.13)	78.12 (75.92, 88.52)^§^	74.97 (70.25, 82.53)	77.49 (70.88, 81.90)	77.49 (74.34, 83.16)	ND
	Control	78.12 (74.97, 91.04)	60.48 (26.46, 74.34)	60.48 (55.76, 75.92)	70.56 (64.89, 75.60)	67.41 (62.69, 103)	ND
IL-10	Allo	257.00 (256.40, 284.80)	268.10 (248.10, 353.30)	271.50 (266.30, 277.70)	257.00 (248.90, 293.30)	270.00 (265.50, 284.80)	ND
	Auto	262.70 (243.50, 280.00)	282.90 (252.60, 293.30)	259.60 (252.90, 292.30)	258.90 (256.40, 321.60)	259.60 (249.20, 264.00)^!^	ND
	Control	245.10 (236.60, 269.00)	250.10 (232.20, 258.90)	248.90 (243.80, 263.70)	248.90 (241.00, 300.20)	261.50 (243.80, 275.00)	ND
TGF-*β*1 (ng/mL)	Allo	34.92 (18.09, 45.03)	16.63 (9.34, 34.78)	35.19 (14.34, 48.16)	41.06 (36.19, 49.19)	48.56 (38.38, 53.50)^#^	69.81 (32.75, 106.90)
	Auto	23.94 (16.25, 36.75)	47.94 (34.94, 76.07)^∗^	40.44 (32.00, 65.50)	39.69 (28.82, 52.94)	41.56 (32.88, 53.25)	79.81 (25.22, 108.80)^∗^
	Control	22.31 (6.50, 36.82)	45.94 (28.82, 54.00)	47.19 (43.06, 51.13)	51.69 (38.50, 59.56)	50.31 (37.94, 53.94)	13.19 (9.81, 14.88)^#,^^
	Healthy control	9.938 (8.44, 9.93)					
IL-4	Allo	144.30 (136.10, 144.30)	137.00 (133.60, 146.20)	137.70 (134.00, 146.90)	133.60 (132.60, 138.00)	137.30 (130.40, 145.20)	ND
	Auto	141.80 (128.80, 145.80)	129.20 (120.60, 142.40)	136.70 (129.20, 141.10)	135.50 (123.50, 143.00)	132.90 (126.00, 134.80)	ND
	Control	127.30 (121.00, 137.70)	132.30 (53.24, 135.80)	130.40 (122.20, 140.20)	139.20 (117.50, 153.40)	129.20 (115.90, 139.50)	ND
IL-6	Allo	175.80 (144.30, 176.40)	124.40 (114.50, 142.40)	150.90 (140.30, 168.50)	131.00 (122.20, 182.10)^§§^	133.60 (132.00, 149.00)^§§^	ND
	Auto	141.10 (122.90, 186.50)	108.40 (104.00, 124.70)	120.30 (116.90, 196.40)	127.30 (119.40, 140.80)^∗∗^^,!!^	123.50 (113.40, 128.20)^∗∗^^,!!^	ND
	Control	159.40 (123.50, 163.50)	117.20 (59.54, 161.00)	117.80 (113.40, 166.30)	110.90 (94.82, 116.20)	107.10 (104.90, 122.90)	ND
Serum creatinine (Scr) (mg/dL)	Allo	9.6 (6.7, 10.3)	1.3 (1.17, 1.67)^∗^	1.18 (0.42, 1.50)^∗^	1.10 (0.77, 1.55)^∗^	1.30 (1.23, 1.45)^∗^	1.25 (1.10, 1.78)^∗^
	Auto	7.6 (6.8, 9.65)	1.40 (1.31, 1.77)^∗^	1.60 (1.22, 1.90)^∗^	1.33 (1.20, 1.71)^∗^	1.40 (1.13, 1.80)^∗^	1.20 (1.10, 1.62)^∗^
	Control	9.3 (8.9, 11.45)	1.44 (1.30, 1.50)^∗^	1.00 (0.75, 1.44)^∗^	1.18 (1.05, 1.36)^∗^	1.20 (1.01, 1.75)^∗^	1.12 (1.01, 1.64)^∗^
	Healthy control	0.85 (0.80, 0.90)					
Estimated glomerular filtration rate (eGFR) (mL/min/1.73m^2^)	Allo	6 (6, 11)	76 (55, 87)^∗^	87 (64, 202)^∗^	95 (60, 127)^∗^	78 (62, 92)^∗^	74 (51, 92)^∗^
	Auto	8 (6, 10)	52 (56, 74)^∗^	59 (49, 71)^∗^	69 (56, 75)^∗^	70 (53, 77)^∗^	85 (52, 92)^∗^
	Control	7 (4, 8)	64 (50, 71)^∗^	85 (62, 116)^∗^	78 (60, 90)^∗^	63 (54, 86)^∗^	74 (50, 94)^∗^
	Healthy control	94 (87, 100)					

Note: values are provided as median (interquartile range). Wilcoxon rank sum test was used to test the differences between auto, allo, control, and healthy control groups. ND: not determined. Analysis within the groups: (a) significant differences from baseline are denoted as ^∗^*p* value ≤ 0.05 and ^∗∗^*p* value ≤ 0.005; (b) significant differences from day 30 are denoted as ^#^*p* value ≤ 0.05 and ^##^*p* value ≤ 0.005; and (c) significant differences from day 90 are denoted as ^^^*p* value ≤ 0.05 and ^^^^*p* value ≤ 0.005. Analysis between the groups: (a) significant differences in comparison to the allo group are denoted as ^!^*p* value ≤ 0.05 and ^!!^*p* value ≤ 0.005; (b) significant differences in comparison to the control group are denoted as ^§^*p* value ≤ 0.05 and ^§§^*p* value ≤ 0.005; and (c) significant differences in comparison to the healthy control group are denoted as ^¶^*p* value ≤ 0.05 and ^¶¶^*p* value ≤ 0.005.

**Table 3:** Studies reporting the use of MSCs in kidney transplant patients (our study summary is at the end column).

Reference no.	Source	Dosage and route of administration	Patient number and groups	Immunosuppressive drugs	Follow-up period	Impact of MSC infusion
Perico et al. [24]	Autologous BM-MSCs	1.7 − 2.0 × 10^6^ cells/kg MSCs were administered intravenously 7 days after KTx	*n* = 2	Induction: basiliximab and low-dose ATG Maintenance: CsA, MMF, and steroids	1 year	Increase in Tregs Inhibition of the memory T cell Reduction of CD8^+^ T cell activity

Tan et al. [25]	Autologous BM-MSCs	1 − 2 × 10^6^/kg of MSCs at kidney reperfusion and intravenously two weeks post-Tx	*n* = 159 Group A = standard-dose CNIs+SCs Group B = received low-dose CNIs+MSCs Group C = control group received anti-IL-2R antibody+standard CNIs	TAC or CsA, MMF, and corticosteroids	1 year	Lower incidence of acute rejection Decreased risk of opportunistic infection Better estimated renal function at 1 year

Reinders et al. [26]	Autologous BM-MSCs	Two doses of 1–2 × 10^6^ cells/kg of MSCs (7 days apart) 6 months after KTx were given to patients with subclinical rejection	*n* = 6	Induction: basiliximab Maintenance: CNI (TAC or CsA), MMF, and prednisone	24 weeks	Patients displayed a downregulation of the mononuclear cell proliferation assay No change in T cells, B cells, NK cells, and monocytes

Perico et al. [27]	Autologous BM-MSCs	2.0 × 10^6^ cells/kg MSCs infused intravenously one day pre-Tx	*n* = 2	Induction: low-dose ATG Maintenance: CsA, MMF, and steroids	1 year	Reduced memory CD8^+^ T cells Low donor-specific CD8^+^ T cell cytolytic response High Treg cells

Vanikar et al. [28]	Adipose-MSCs (AD-MSCs) and BM-HSCs	0.04 × 10^6^ MSCs/kg+8-10 × 10^8^ HSCs/kg 5 days before Tx through portal infusion	*n* = 285 Group1 = AD-MSC+HSCs+drugs Group 2 = HSC+drugs Group 3 = drugs only	ATG, total lymphocyte irradiation, TAC, MEP	5-7 years	Better graft survival in groups 1 and 2

Pan et al. [29]	Donor-derived, BM-MSCs	5 × 10^6^ MSCs were infused using a pressurizer during KTx 2 × 10^6^ cells/kg were administered intravenously after 30 days of KTx	*n* = 32 MSC group and non-MSC group	Induction: cytoxan and methylprednisolone Maintenance: TAC, MMF, and prednisone	2 years	Low-dose TAC and MSCs were as effective as standard-dose TAC in graft survival after transplantation No differences in CD19, CD3, CD34, CD38, and NK cells were detected

Perico et al. [30]	Autologous BM-MSCs	1.7 − 2.0 × 10^6^ cells/kg MSCs were given intravenously at day 7 post-Tx (*n* = 2) or at day 1 pre-Tx (*n* = 2)	*n* = 4	Induction: basiliximab and low-dose ATG Maintenance: low-dose CsA, MMF	5-7 years	Low circulating memory CD8^+^ T cells (*n* = 3) Reduction of ex vivo donor-specific cytotoxicity (*n* = 3) Increase in the Treg cell/memory CD8^+^ T cell ratio High circulating levels of naïve and transitional B cells (*n* = 2)

Sun et al. [31]	Human umbilical cord-derived MSCs (UC-MSCs)	2 × 10^6^/kg of MSCs via the peripheral vein before KTx 5 × 10^6^ cells via the renal artery during KTx	*n* = 42 MSC group and non-MSC group	Induction: ATG and methylprednisolone Maintenance: CNI (TAC or CsA), MMF, and prednisone	1 year	UC-MSCs can be used as clinically feasible and safe induction therapy

Erpicum et al. [32]	Third-party bone marrow- derived MSCs (BM-MSCs)	∼2 × 10^6^/kg of MSCs centrally infused on day 3 ± 2 post-KTx	*n* = 20 MSC group and non-MSC group	TAC, MMF, corticosteroids	1 year	Increased Treg frequencies No significant change in B cell frequencies

Casiraghi et al. [7]	Autologous BM-MSCs	2 × 10^6^/kg MSCs intravenously, one day before KTx	*n* = 1 (case study)	Induction: low-dose ATG, D-0 to D-6 after KTx Maintenance: CsA, MMF, methylprednisolone	9 years	Increased Tregs Reduced memory CD8^+^ T cells Increased naïve B cells and transitional B cells Safe withdrawal of maintenance drugs

Kaundal et al. [6]	Autologous BM-MSCs	Two doses of 1-1.5 × 10^6^/kg MSCs intravenously, one day before and 30 days after KTx	*n* = 10 Auto group and control group	TAC, MMF, methylprednisolone	2 years	Decrease in B cells Increase in the transitional B cell subset

Dreyer et al. [33]	Third-party BM-MSCs	Two doses of 1.5 × 10^6^/kg allogeneic MSCs 6 months post-Tx	*n* = 10	Induction: alemtuzumab Maintenance: prednisone, low-dose TAC, and everolimus		No alterations in T and B cell populations or plasma cytokines HLA-selected allogeneic MSCs combined with low-dose tacrolimus 6 months post-Tx are safe

Kaundal et al. (current manuscript)	Autologous BM-MSCs and allogeneic BM-MSCs	Two doses of 1-1.5 × 10^6^/kg MSCs intravenously, one day before and 30 days after KTx	*n* = 15 Auto group, allo group, and control group	TAC, MMF, methylprednisolone	2 years	Upregulation of naive T subsets and B regulatory and double-negative T cells Clinical parameters normalized including Scr Rejection episodes more in infused groups

## Data Availability

Supporting data were available as supplementary figures.

## Supplementary Materials

Supplementary Materials Supplementary Tables (ST 1 to ST 7). ST 1: patient eligibility criteria. ST 2: primary and secondary study objectives and endpoints. ST 3: patient demographics and clinical profile of renal transplant patients. ST 4: cell characteristics postmanufacturing. ST 5: statistical summary of the clinical parameters in study groups. ST 6: resource table. ST 7: HLA typing of the renal recipient and donor. Supplementary Figures (SF 1 to SF 4). SF 1: CONSORT flow diagram: trial reporting for screening, enrollment, allocation, follow-up, and analysis for MSC infusion in autologous (auto) and allogeneic (allo) groups along with the control group. SF 2: MSC characterization of bone marrow-derived mesenchymal stromal cells. (A) Representative light microscopy picture of spindle-shaped adherent MSCs (P-2) (magnification: 20x). (B) MSC gating according to the FSC and SSC profile. Flow cytometric analysis indicated that BM-MSCs are negative for CD34, CD45, CD11b, CD19, and HLA-DR (negative cocktail) and positive for MSC surface markers CD73, CD90, and CD105. Dark grey-coloured plots represent specific antibody staining, and light grey plots represent negative control. (C) Representative images depicting *in vitro* differentiation assays revealing formation of lipid droplets stained with Oil Red O (20x, formation of chondrocytes stained with Alcian Blue (40x), formation of osteocytes stained with Alizarin Red S (20X). (D) Representative normal complete karyogram -46, XX, of culture-expanded MSCs at passage 3. Karyotypic analysis was done for all samples of expanded MSCs that were used for infusions. (E) Sterility testing was performed for all samples used for infusion. Anaerobic bacterial, aerobic bacterial, mycoplasma, and fungal contamination was ruled out before the infusion. SF 3: representative figures depicting the gating strategy for the identification of human T cell subsets. (A) Lymphocyte gating according to the FSC and SSC profile. Lymphocytes were then gated to determine the proportion of (B) CD3^+^ cells, (C) CD4^+^ T cells, CD8^+^ T cells, and CD4^−^CD8^−^ T (double-negative (DN) T) cells. For detecting effector and memory cell subsets, first (D) CD4^+^CD45RO^+^CD45RA^−^ and CD4^+^CD45RO^−^CD45RA^+^ cells and (E) CD8^+^CD45RO^+^CD45RA^−^ and CD8^+^CD45RO^−^CD45RA^+^ cells were gated, and then, on the basis of CD62L staining, the cells were identified as (F) CD4 central memory (CD4 T_MEM-CM_) and CD4 effector memory cells (CD4 T_MEM-EM_), (G) CD4 naive (CD4 T_NAI_) and CD4 effector (CD4 T_EFF_) cells, (H) CD8 central memory (CD8 T_MEM-CM_) and CD8 effector memory cells (CD8 T_MEM-EM_), and (I) CD8 naive (CD8 T_NAI_) and CD8 effector cells (CD8 T_EFF_). (J) Lymphocyte gating to determine the proportion of (K) CD3^+^CD4^+^ T cells. Further from CD3^+^CD4^+^ T cells, (L) CD25^hi^ cells were gated for the (M) T_REGS_ (FoxP3^+^ CD127^lo^) population. FCS-A: forward scatter area; SSC-A: side scatter area. SF 4: representative figures depicting the gating strategy for the identification of human B cell subsets. (A) Lymphocyte gating according to the FSC and SSC profile. Lymphocytes were then gated to determine the proportion of (B) CD19^+^ B cells, which on the basis of CD38 and IgD double staining were further classified as (C) virgin naive (bm1) IgD^+^CD38^−^, activated naive (bm2) IgD^+^CD38^+^, pregerminal (bm2′) IgD^+^CD38^hi^, germinal center (bm3^+^ bm4) IgD^−^CD38^hi^, early memory (ebm5) IgD^−^CD38^+^, and late memory (bm5) IgD^−^CD38^−^ cells. On the basis of IgD-CD27 double staining, CD19^+^ B cells were also identified as (D) switched B cells—IgD^−^CD27^+^; unswitched B cells—IgD^+^CD27^+^; naive (B_NAI_)—IgD^+^CD27^−^; and double negative (DN B) IgD^−^CD27^−^ cells. Regulatory B cell subsets were determined from the CD19^+^ B cells by gating (E) CD5^+^ cells and (F) CD1d^hi^ cells for the B_REG_ subset, (G) CD27^+^ cells and (H) CD24^hi^ for B_10_ cells, and (I) CD24^hi^ cells and (J) CD38^hi^ cells for immature B (B_IM_) cells. FCS-A: forward scatter area; SSC-A: side scatter area.
