# Supplementary material for: Mesenchymal Stromal Cells Mediate Clinically Unpromising but Favourable Immune Responses in Kidney Transplant Patients
Source: Stem Cells Int. 2022 Feb 15;2022:2154544. doi: 10.1155/2022/2154544 (PMC8863486; doi:10.1155/2022/2154544)
Supplement: Supplementary Materials — Supplementary Tables (ST 1 to ST 7). ST 1: patient eligibility criteria. ST 2: primary and secondary study objectives and endpoints. ST 3: patient demographics and clinical profile of renal transplant patients. ST 4: cell characteristics postmanufacturing. ST 5: statistical summary of the clinical parameters in study groups. ST 6: resource table. ST 7: HLA typing of the renal recipient and donor. Supplementary Figures (SF 1 to SF 4). SF 1: CONSORT flow diagram: trial reporting for screening, enrollment, allocation, follow-up, and analysis for MSC infusion in autologous (auto) and allogeneic (allo) groups along with the control group. SF 2: MSC characterization of bone marrow-derived mesenchymal stromal cells. (A) Representative light microscopy picture of spindle-shaped adherent MSCs (P-2) (magnification: 20x). (B) MSC gating according to the FSC and SSC profile. Flow cytometric analysis indicated that BM-MSCs are negative for CD34, CD45, CD11b, CD19, and HLA-DR (negative cocktail) and positive for MSC surface markers CD73, CD90, and CD105. Dark grey-coloured plots represent specific antibody staining, and light grey plots represent negative control. (C) Representative images depicting in vitro differentiation assays revealing formation of lipid droplets stained with Oil Red O (20x, formation of chondrocytes stained with Alcian Blue (40x), formation of osteocytes stained with Alizarin Red S (20X). (D) Representative normal complete karyogram -46, XX, of culture-expanded MSCs at passage 3. Karyotypic analysis was done for all samples of expanded MSCs that were used for infusions. (E) Sterility testing was performed for all samples used for infusion. Anaerobic bacterial, aerobic bacterial, mycoplasma, and fungal contamination was ruled out before the infusion. SF 3: representative figures depicting the gating strategy for the identification of human T cell subsets. (A) Lymphocyte gating according to the FSC and SSC profile. Lymphocytes were then gated to determine the [file 2154544.f1.zip › Supplementary Information_revised (1).pdf]

SF 1

CONSORT
TRANSPARENT REPORTING of TRIALS

CONSORT 2010 Flow Diagram

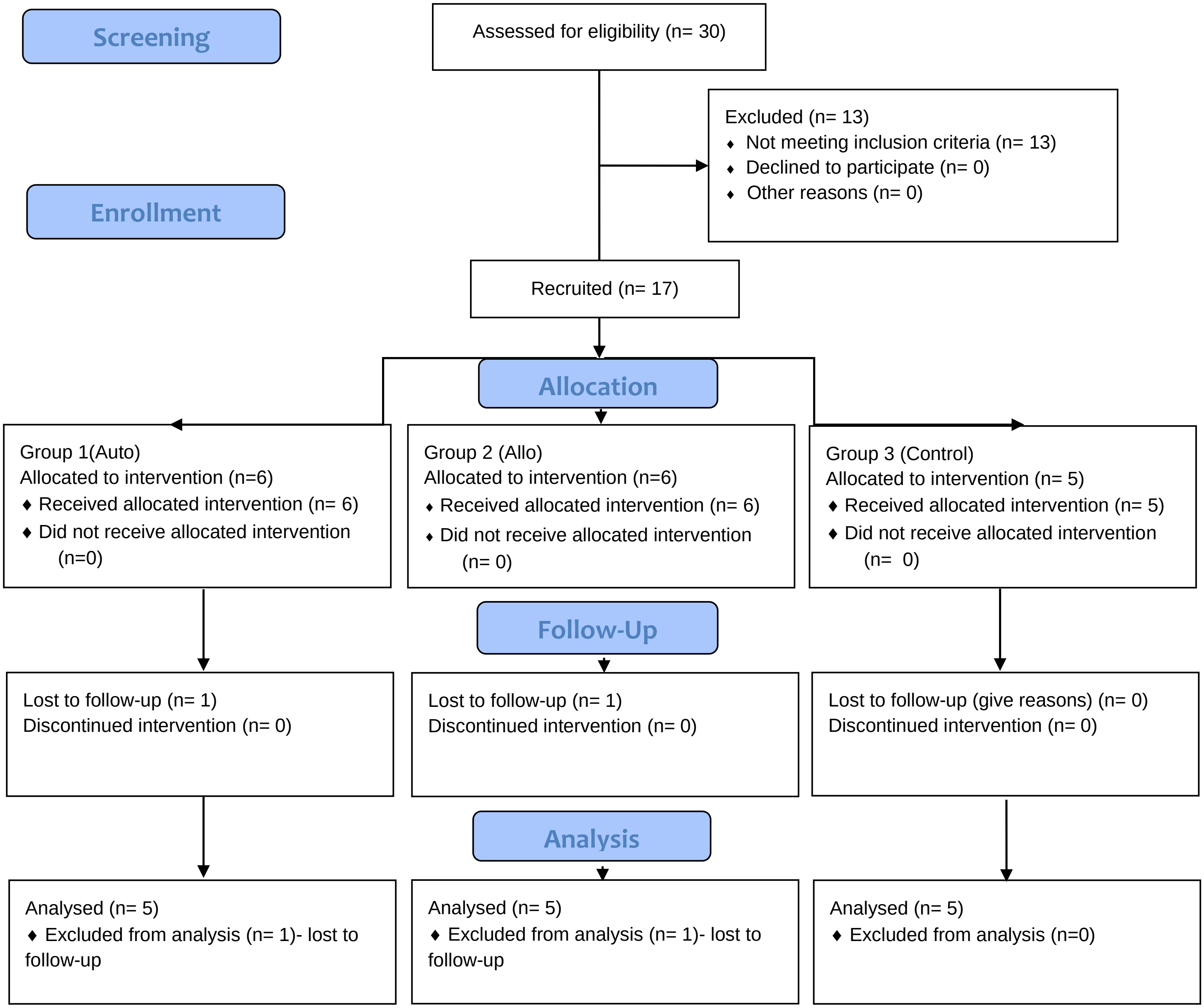

SF 2

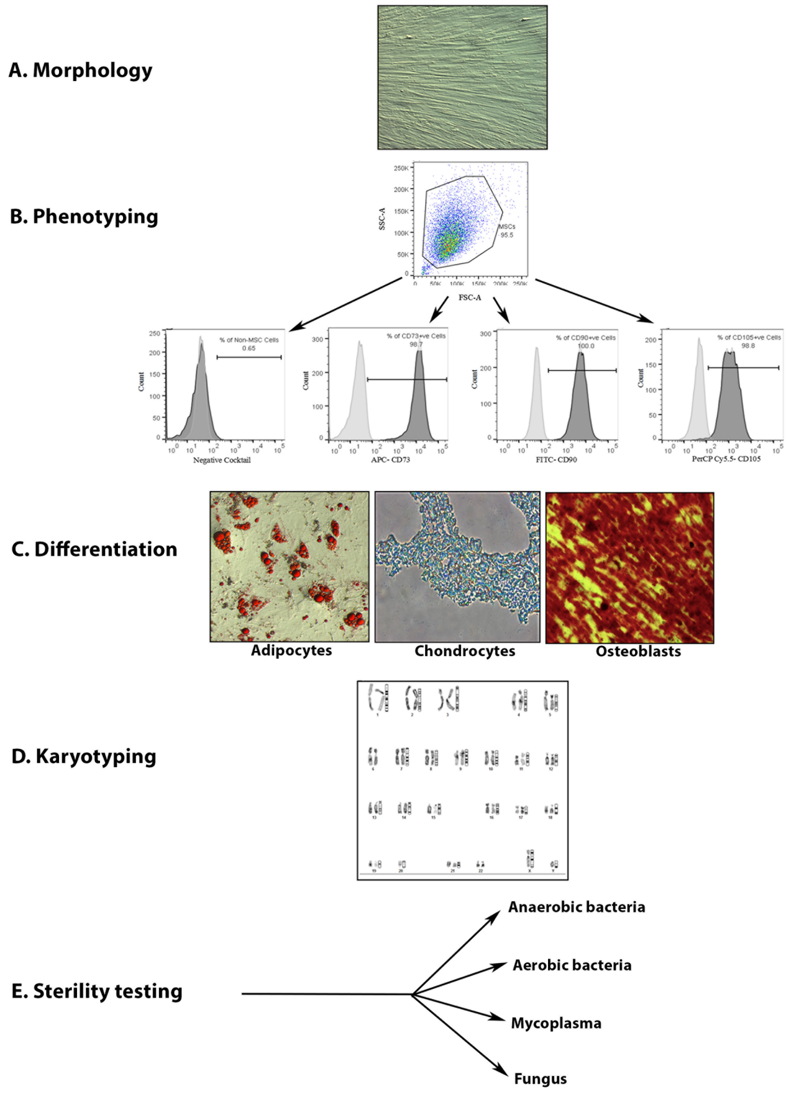
A. Morphology
B. Phenotyping
SSC-A
FSC-A
Negative Cocktail
APC- CD73
FITC- CD90
PerCP Cy5.5- CD105
C. Differentiation
Adipocytes
Chondrocytes
Osteoblasts
D. Karyotyping
E. Sterility testing
Anaerobic bacteria
Aerobic bacteria
Mycoplasma
Fungus

SF 3

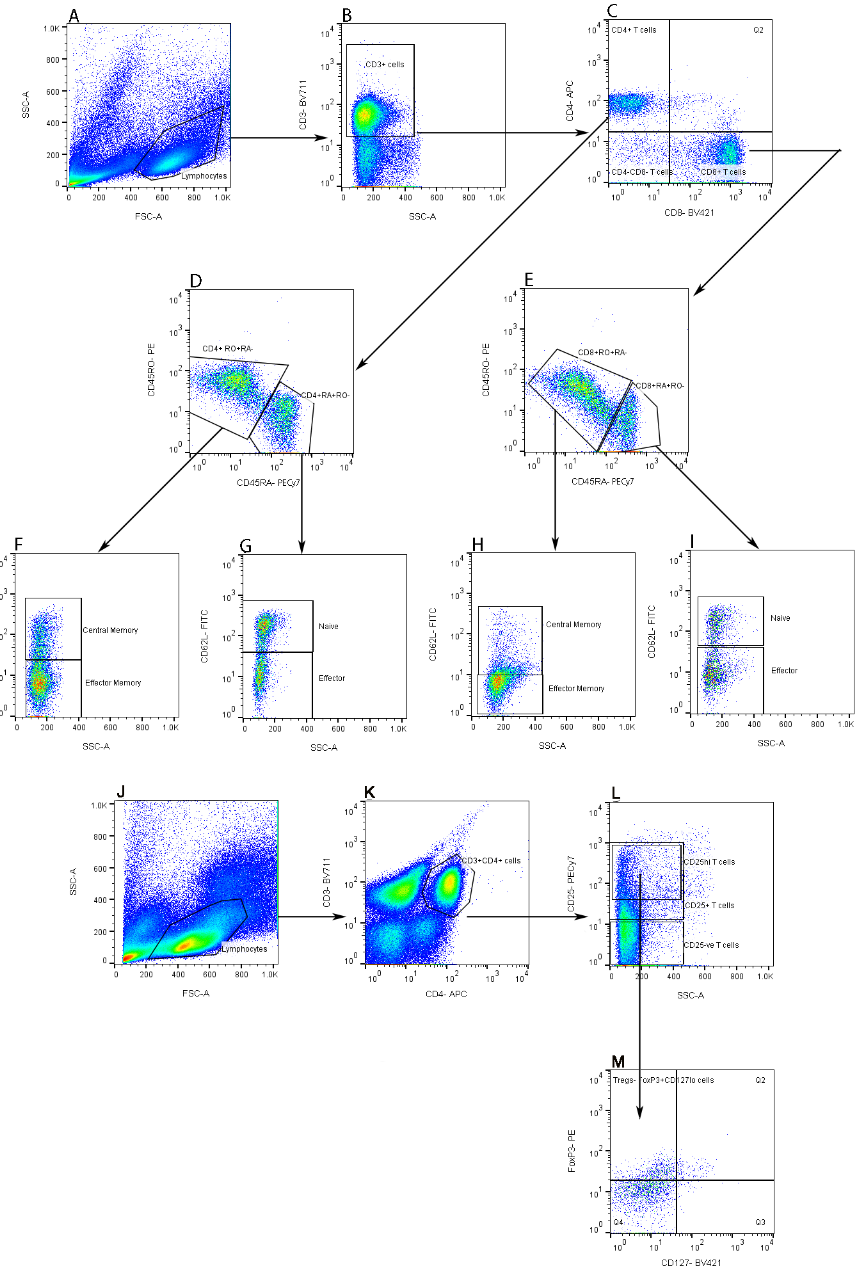

A
Lymphocytes
SSC-A
FSC-A
B
CD3+ cells
CD3- BV711
SSC-A
C
CD4+ T cells
Q2
CD4-CD8- T cells
CD8+ T cells
CD4- APC
CD8- BV421
D
CD4+ RO+RA-
CD4+RA+RO-
CD45RO- PE
CD45RA- PECy7
E
CD8+RO+RA-
CD8+RA+RO-
CD45RO- PE
CD45RA- PECy7
F
Central Memory
Effector Memory
CD62L- FITC
SSC-A
G
Naive
Effector
CD62L- FITC
SSC-A
H
Central Memory
Effector Memory
CD62L- FITC
SSC-A
I
Naive
Effector
CD62L- FITC
SSC-A
J
Lymphocytes
SSC-A
FSC-A
K
CD3+CD4+ cells
CD3- BV711
CD4- APC
L
CD25hi T cells
CD25+ T cells
CD25-ve T cells
CD25- PECy7
SSC-A
M
Q2
Q4
Q3
FoxP3- PE
CD127- BV421

SF 4

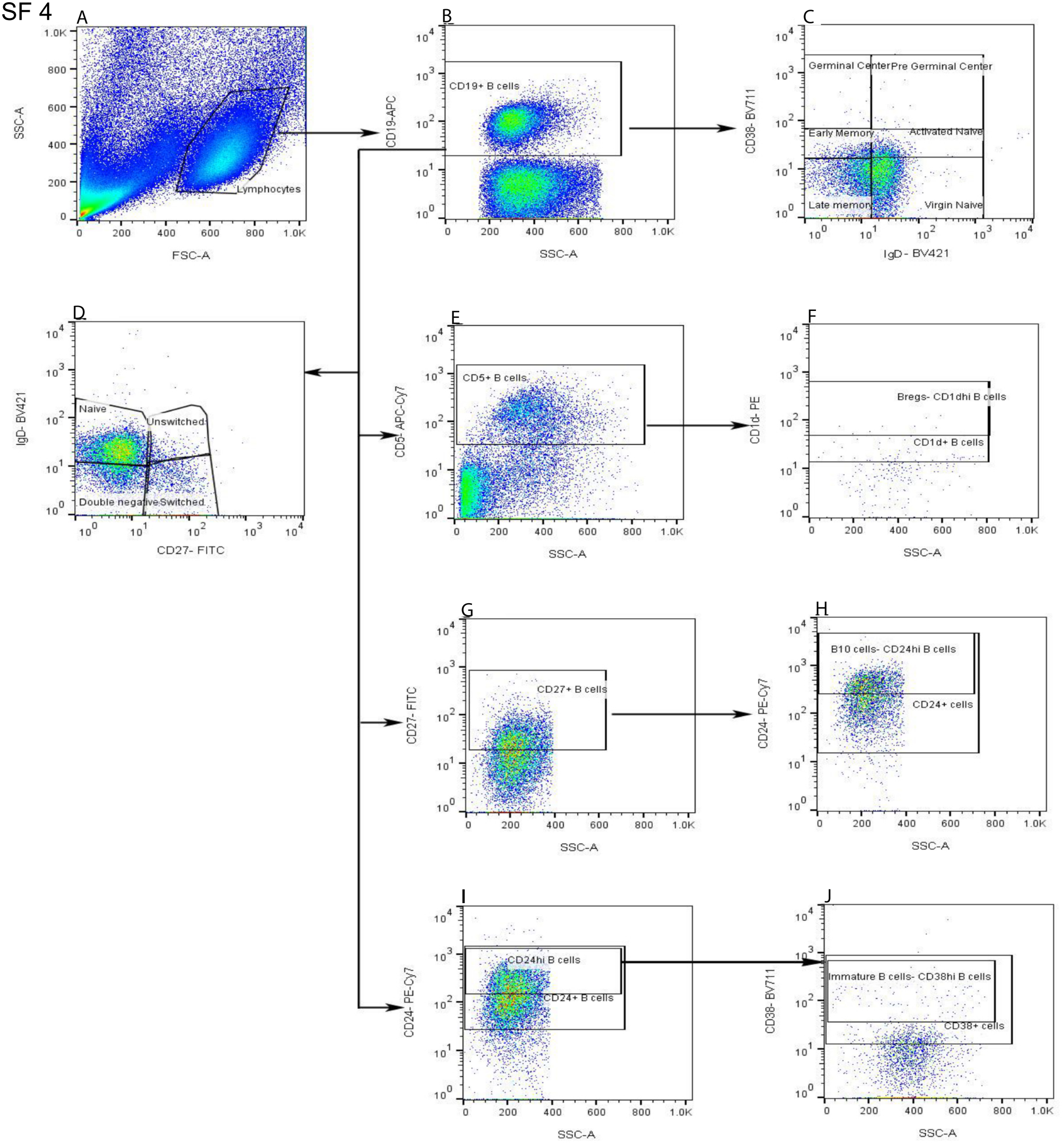

A
SSC-A
FSC-A
Lymphocytes
B
CD19-APC
CD19+ B cells
SSC-A
C
CD38- BV711
Germinal Center
Pre Germinal Center
Early Memory
Activated Naive
Late memory
Virgin Naive
IgD- BV421
D
IgD- BV421
Naive
Unswitched
Double negative
Switched
CD27- FITC
E
CD5- APC-Cy7
CD5+ B cells
SSC-A
F
CD1d- PE
Bregs- CD1dhi B cells
CD1d+ B cells
SSC-A
G
CD27- FITC
CD27+ B cells
SSC-A
H
CD24- PE-Cy7
B10 cells- CD24hi B cells
CD24+ cells
SSC-A
I
CD24- PE-Cy7
CD24hi B cells
CD24+ B cells
SSC-A
J
CD38- BV711
Immature B cells- CD38hi B cells
CD38+ cells
SSC-A

Table ST 1: Patient Eligibility Criteria

**Inclusion Criteria**
- Histopathologically confirmed cases of end stage renal disease (ESRD).<br>- undergoing renal transplant with kidney donated by a living related donor.<br>- Written informed consent.
**Exclusion Criteria**
- Patient who has received any kind of solid organ transplant<br>- Patient with unrelated or cadaveric donors<br>- Receiving ATG as a part of IS regime<br>- Incidence of Human immunodeficiency virus (HIV), hepatitis C virus (HCV) or any other secondary infections<br>- Low hemoglobin or any blood-related disorders<br>- History of kidney disease inflicted by urinary tract infections.

Table ST 2: Primary and Secondary Study Objectives and Endpoints

**Primary Objectives**
The primary objective of this study was to assess safety and feasibility of autologous and donor-derived (allogeneic) mesenchymal stem cells in combination with immunosuppressive regime (TAC+MMF+Prednisolone) in living-donor KTx patients (n=12 (n=6 in auto group & n=6 in allo group). Results were compared to the control group (n=5) which included patients who didn't receive any MSCs to determine the differences in the immune profiles.
**Primary Safety Endpoints**
To assess safety and feasibility of MSC infusions in kidney transplant patients on the basis of:<br>- Graft rejection/failure<br>- Adverse events post-MSC infusion<br>- Opportunistic infections due to immunosuppressive therapy
**Secondary Objective**
To evaluate the effect of MSC infusion on the immune profile of kidney transplant patients in comparison to the control group.
**Secondary Endpoints**
Immunological markers associated with common immune responses.

ST 3: Patient Demographics, Clinical Profile of Renal Transplant Patients

Patients from groups	Age/Gender of patient	Body Weight (Kgs.)	Age/Gender of donor	Relationship with donor	Cause of CKD	HLA Match	Follow up duration	CMV serostatus	Rejection episodes/ Banff classification	Diabetes	Anaemia	Opportunistic Infections
P1 (allo)	19/M	33	40/F	Mother	*	4/4	2 years	+ve	No	No	No	No
P3 (allo)	31/M	50	57/F	Mother	AN	2/4	2 years	+ve	Borderline Acute TCMR at 3.5 months Category 3 t_2 i_1	No	No	No
P4 (allo)	32/M	56	48/F	Mother	Steroid resistant FSGS	2/4	2 years	+ve	No	No	No	No
P5 (allo)	22/M	37	62/F	Mother	IgAN	2/4	2 years	+ve	No	No	No	No
P6 (allo)	42/M	46	48/M	Brother	CGN/CRF	3/6	2 years	+ve	Acute TCMR immediately after Tx Category 4 $v_3t_3i_3$	Yes	No	No
Pa1 (auto)	28/M	51	55/F	Mother	CGN/CRF	2/4	2 years	+ve	No	No	Yes	No
Pa2 (auto)	24/M	61	45/F	Mother	HN	3/4	2 years	+ve	No	No	No	No
Pa3 (auto)	23/M	51	49/F	Mother	CGN/CRF	2/4	2 years	+ve	No	No	No	No
Pa5 (auto)	23/M	55	55/M	Father	RPGN	2/4	2 years	+ve	Acute ABMR immediately after Tx Category 2 ptc_3/g_3	No	No	No
Pa6 (auto)	26/F	55.3	35/F	Sister	CGN/CRF	3/4	2 years	+ve	Mixed Rejection immediately after Tx Acute ABMR, Category 2, ptc_3/g_3 + Borderline Acute TCMR, Category 3, t_1 i_1	No	No	No
C3 (control)	26/M	52	34/F	Sister	CGN/CRF	*	2 years	+ve	No	No	No	Yes
C4 (control)	25/M	49	47/F	Mother	CGN/CRF	2/4	2 years	+ve	No	No	No	No
C5 (control)	44/F	60	45/F	Sister	HN	4/6	2 years	+ve	No	No	No	Yes
C7 (control)	25/F	48	46/F	Mother	CGN/CRF	3/6	2 years	+ve	No	No	No	No
C8 (control)	22/M	56	48/F	Mother	CGN/CRF	3/6	2 years	+ve	No	No	No	No

TCMR – T cell mediated rejection, ABMR – Antibody mediated rejection, AN-Analgesic nephropathy; FSGS- Focal segmental glomerulosclerosis; IgAN- IgA nephropathy; CGN/CRF- Chronic glomerulonephritis/Chronic Renal Failure ; HN- Hypertensive nephrosclerosis; RPGN- Progressive glomerulosclerosis, t-tubulitis, i- interstitial inflammation, ptc – Peritubular Capillaritis, g- Glomerulitis
* Data could not be retrieved

Table ST 4. Cell Characteristics Post Manufacturing

Grp#	Patients	Body Weight	First Dose (Day-0 pre-transplant)						Second Dose (Day-30 post-transplant)					
		(Kg.)	Cell Number ($X10^6$) (P 2)	Cells/Body Weight ($X10^6$/kg)	Purity ($CD34^-$ $CD45^-$ $CD73^+$ $CD90^+$ $CD105^+$)	Viability (Trypan Blue)	Aerobic, Anaerobic, Mycoplasma Testing	Karyotyping	Cell Number ($X10^6$) (P 2)	Cells/Body Weight ($X10^6$/kg)	Purity ($CD34^-$ $CD45^-$ $CD73^+$ $CD90^+$ $CD105^+$)	Viability (Trypan Blue)	Aerobic, Anaerobic, Mycoplasma Testing	Karyotyping
Auto	Pa1	51	120	2.35	>95%	>98%	No contamination	No abnormality	128	2.50	>95%	>98%	No contamination	No abnormality
	Pa2	61	85	1.39	>95%	>98%	No contamination	No abnormality	80	1.31	>95%	>98%	No contamination	No abnormality
	Pa3	51	110	2.15	>95%	>98%	No contamination	No abnormality	105	2.05	>95%	>98%	No contamination	No abnormality
	Pa5	55	69.5	1.26	>95%	>98%	No contamination	No abnormality	*	*	>95%	>98%	No contamination	No abnormality
	Pa6	55.3	60	1.08	>95%	>98%	No contamination	No abnormality	*	*	>95%	>98%	No contamination	No abnormality
Allo	P1	33	58	1.7	>95%	>98%	No contamination	No abnormality	62	1.87	>95%	>98%	No contamination	No abnormality
	P3	50	92	1.84	>95%	>98%	No contamination	No abnormality	83	1.66	>95%	>98%	No contamination	No abnormality
	P4	56	88	1.57	>95%	>98%	No contamination	No abnormality	80	1.42	>95%	>98%	No contamination	No abnormality
	P5	37	75	2.02	>95%	>98%	No contamination	No abnormality	70	1.89	>95%	>98%	No contamination	No abnormality
	P6	46	77	1.67	>95%	>98%	No contamination	No abnormality	98	2.13	>95%	>98%	No contamination	No abnormality

* Second infusion was not done for these patients
# Group

Table ST 5: Statistical summary of the clinical parameters in study groups

Patient parameters	Normal Range and units	Group (n=5 in each group)	Baseline	Day-30	Day-90	Day-180	Day-365	Day-800	Kruskal Wallis test (p-value)
**Weight**	Kg	**Allo**	44.0±9.1	42.3±12.5	44.6±14.6	55.5±5.3	52.7±7.7	49.2±14.5	0.38
		**Auto**	49.0±2.1	51.8±2.3	52.8±7.0	53.6±7.0	58.6±6.0	55.2±8.4	0.53
		**Control**	48.25±4.4	53.0±4.2	47.0±7.0	42.5±0.70	55.2±11.3	51.25±13.6	0.68
**TLC**	4000-11000 cells/µL	**Allo**	7420±3795	10367±611	8300±2401	9933±1856	10800±964	10950±1061	0.33
		**Auto**	7640±3388	9250±3308	8325±1895	6860±2514	9250±3748	9000±2546	0.75
		**Control**	12380±2535	13500±3387	8100±0	7000±0	8525±854	10067±2503	0.07
**Total protein**	6-8.3 g/dL	**Allo**	6.7±0.8	7.0±0.1	6.16±0.22	6.9±0.49	7.1±1.4	6.38±0.27	0.55
		**Auto**	6.2±0.6	6.9±0.2	6.5±0.6	6.68±0.02	7.5±0.1	7.2±0.39	0.23
		**Control**	6.05±0.4	6.9±0.2	7.2±0.7	7.1±0.4	6.8±0.09	6.6±0.36	**0.02**
**Albumin**	3.5-5.2 g/dL	**Allo**	4.1±0.9	4.3±0.1	4.7±0.1	4.5±0.1	4.5±0.1	4.8±0.1	0.36
		**Auto**	3.7±0.4	4.3±0.4	4.5±0.1	4.2±0.1	4.1±0.1	4.4±0.2	0.30
		**Control**	4.2±0.3	4.0±0	4.4±0	4.7±0.2	3.9±.1	3.8±0.5	0.11
**Bilirubin**	0.2-1.2 mg/dL	**Allo**	0.3±0.2	0.4±0.1	0.2±0.1	0.5±0.1	0.7±0.5	0.6±0.5	0.24
		**Auto**	0.6±0.2	0.3±0.1	0.4±0.0	0.4±0.3	0.6±0.1	0.8±0.1	0.21
		**Control**	0.5±0.3	0.3±0.03	-	1.1±0	0.76±0.65	0.9±0.4	0.27
**AST**	0-40 (U/I)	**Allo**	17.8±8.1	23.7±8.4	19.3±8.0	23.3±2.5	22.0±3.9	20.6±6.3	0.64
		**Auto**	31.2±8.5	27.4±8.3	30±12.57	28±4.7	22.5±13.4	29±5.6	0.95
		**Control**	19.4±5.3	29.5±3.5	24.0±5.6	21.0±4.2	24.0±8.0	27.0±14.0	0.57
**ALT**	0-31 (U/I)	**Allo**	22.6±10.1	18.7±1.2	15.0±4.3	31.7±14.6	19.2±6.2	25.3±6.6	0.36
		**Auto**	27.2±13.9	28.2±14.4	25.6±6.8	29.3±25.7	16±0.1	25±7.7	0.93
		**Control**	21.2±12.6	68±0	22.0±5.6	27.7±12.1	26.3±7.2	20.0±2.6	0.48
**Alkaline phosphatase**	27-90 (U/I)	**Allo**	86.5±7.7	66.0±8.4	74.5±27.5	69.3±25.7	79.0±0.1	85.0±26.8	0.83
		**Auto**	82.7±19.0	89.3±5.5	87±0.1	78.6±7.0	87.0±0.1	71.5±4.9	0.51
		**Control**	88±2.8	138.5±62.9	200±14.1	170±0.0	328±258	362.5±167.6	0.11
**Calcium**	8.6-10.0 (mg/dL)	**Allo**	8.6±1.2	8.9±0.5	8.7±0.2	8.8±0.3	8.6±0.8	8.8±0.8	0.89
		**Auto**	8.8±0.4	9.5±0.1	10.0±0.1	8.7±1.3	9.4±0.1	9.5±0.7	0.25
		**Control**	9.6±0.6	10.2±1.1	9.3±0.0	9.01±0.83	7.8±0.0	8.6±0.2	0.30

µl- microliter, g/dL- grams per decilitre, mg/dL- milligram per decilitre, AST- aspartate aminotransferase, ALT- alanine aminotransferase. n=5 in each group.

Table ST 6: Resource table

**REAGENTS**	**SOURCE**
**Antibodies**	
CD3-BV711	BD Biosciences
CD4-APC	BD Biosciences
CD8-BV421	BD Biosciences
CD45RO-PE	BD Biosciences
CD45RA-PE-CyTM 7	BD Biosciences
CD62L-FITC	BD Biosciences
CD25-PE-Cy^{TM} 7	BD Biosciences
CD127-BV421	BD Biosciences
FoxP3-PE	BD Biosciences
CD19-APC	BD Biosciences
CD1d-PE	BD Biosciences
CD5-APC-Cy^{TM} 7	BD Biosciences
CD24-PE-Cy^{TM} 7	BD Biosciences
CD27-FITC	BD Biosciences
CD38-BV711	BD Biosciences
IgD-BV421	BD Biosciences
CD73-APC	BD Biosciences
CD90-FITC	BD Biosciences
CD105-PerCP-Cy5.5	BD Biosciences
MSC-Negative cocktail-PE	BD Biosciences
Purified CD3	BioLegend
Purified CD28	BioLegend
**Cytokines/Factors detection kits**	
Th1/Th2/Th17 CBA kit (IL-2, 4, 6, 10, 17A TNF-α, IFN-γ)	BD Biosciences
TGF-β1	DRG®

Table ST 7: HLA Typing of Renal Recipient and Donor

Patient ID	HLA Recipient	HLA Donor
**P1**	A24 A33 B22(55) BW4	A24 A33 B22(55) BW 4
**P3**	A24 (9) A11 B27 B40	A24 (9) A31 B40
**P4**	A1 B35	A1 A33 B35 B52
**P5**	A1 A33 B52(5) B37 BW4	A33 A33 B44(12) B52 DRB1 DR04 DR08
**P6**	A1 A24 B53 B57 DR04 DR07	A01 A33 B57 B58 DRDR DR07 DR11
**Pa1**	A02 A11 B35(5) BW4 BW6	A11 B35(5) B51 B52 BW4 BW6
**Pa2**	A02 B15 B35 BW4 BW6	A02 A11 B15 B35 BW6
**Pa3**	A11 A33 B35 BW 6	A03 A33 B17 B35 BW4 BW6
**Pa5**	A28 B52(5) B55 B22 BW4 BW6	A11 A28 B55(22) B35 B5 BW6
**Pa6**	A02 A03 B51 B18 BW4 BW6	A2 A33 B51 B15 BW4 BW6
**C4**	A02 A33 B44 B40 DR14 DR14	A33 A35 B44 B44 BW7 BW14
**C5**	A11 A24 B35 B40 DR13 DR15	A24 A24 B35 B40 DR13 1DR5
**C7**	A02 A02 B40 B52(5) DR15 DR13	A02 A74 B52 B38 DR15 DR04
**C8**	A02 A31 B15 B15 DR03 DR11	A02 A68 B15 B3 B5 DR11 DR11
