# Supplementary material for: Mesenchymal Stromal Cells Mediate Clinically Unpromising but Favourable Immune Responses in Kidney Transplant Patients
Source: Stem Cells Int. 2022 Feb 15;2022:2154544. doi: 10.1155/2022/2154544 (PMC8863486; doi:10.1155/2022/2154544)
Supplement: Supplementary Materials — Supplementary Tables (ST 1 to ST 7). ST 1: patient eligibility criteria. ST 2: primary and secondary study objectives and endpoints. ST 3: patient demographics and clinical profile of renal transplant patients. ST 4: cell characteristics postmanufacturing. ST 5: statistical summary of the clinical parameters in study groups. ST 6: resource table. ST 7: HLA typing of the renal recipient and donor. Supplementary Figures (SF 1 to SF 4). SF 1: CONSORT flow diagram: trial reporting for screening, enrollment, allocation, follow-up, and analysis for MSC infusion in autologous (auto) and allogeneic (allo) groups along with the control group. SF 2: MSC characterization of bone marrow-derived mesenchymal stromal cells. (A) Representative light microscopy picture of spindle-shaped adherent MSCs (P-2) (magnification: 20x). (B) MSC gating according to the FSC and SSC profile. Flow cytometric analysis indicated that BM-MSCs are negative for CD34, CD45, CD11b, CD19, and HLA-DR (negative cocktail) and positive for MSC surface markers CD73, CD90, and CD105. Dark grey-coloured plots represent specific antibody staining, and light grey plots represent negative control. (C) Representative images depicting in vitro differentiation assays revealing formation of lipid droplets stained with Oil Red O (20x, formation of chondrocytes stained with Alcian Blue (40x), formation of osteocytes stained with Alizarin Red S (20X). (D) Representative normal complete karyogram -46, XX, of culture-expanded MSCs at passage 3. Karyotypic analysis was done for all samples of expanded MSCs that were used for infusions. (E) Sterility testing was performed for all samples used for infusion. Anaerobic bacterial, aerobic bacterial, mycoplasma, and fungal contamination was ruled out before the infusion. SF 3: representative figures depicting the gating strategy for the identification of human T cell subsets. (A) Lymphocyte gating according to the FSC and SSC profile. Lymphocytes were then gated to determine the [file 2154544.f1.zip › SF1.doc]

**CONSORT 2010 Flow Diagram**

**Allocation**

**Analysis**

**Follow-Up**

**Screening**

**Enrollment**

Assessed for eligibility (n= 30)

Excluded (n= 13)

♦  Not meeting inclusion criteria (n= 13)

♦  Declined to participate (n= 0)

♦  Other reasons (n= 0)

Analysed (n= 5)
♦ Excluded from analysis (n= 1)- lost to follow-up

Lost to follow-up (n= 1)
Discontinued intervention (n= 0)

Group 1(Auto)
Allocated to intervention (n=6)

♦ Received allocated intervention (n= 6)

♦ Did not receive allocated intervention (n=0)

Lost to follow-up (give reasons) (n= 0)
Discontinued intervention (n= 0)

Group 3 (Control)
Allocated to intervention (n= 5)

♦ Received allocated intervention (n= 5)

♦ Did not receive allocated intervention (n= 0)

Analysed (n= 5)
♦ Excluded from analysis (n=0)

Recruited (n= 17)

Group 2 (Allo)

Allocated to intervention (n=6)

♦ Received allocated intervention (n= 6)

♦ Did not receive allocated intervention (n= 0)

Lost to follow-up (n= 1)
Discontinued intervention (n= 0)

Analysed (n= 5)
♦ Excluded from analysis (n= 1)- lost to follow-up
